# Phytosome Supplements for Delivering *Gymnema inodorum* Phytonutrients to Prevent Inflammation in Macrophages and Insulin Resistance in Adipocytes

**DOI:** 10.3390/foods12112257

**Published:** 2023-06-03

**Authors:** Onanong Nuchuchua, Ratchanon Inpan, Wanwisa Srinuanchai, Jirarat Karinchai, Pornsiri Pitchakarn, Ariyaphong Wongnoppavich, Arisa Imsumran

**Affiliations:** 1 National Nanotechnology Center (NANOTEC), National Science and Technology Development Agency (NSTDA), Pathum Thani 12120, Thailand; onanong@nanotec.or.th (O.N.); wanwisa.sri@nanotec.or.th (W.S.); 2 Department of Biochemistry, Faculty of Medicine, Chiang Mai University, Chiang Mai 50200, Thailand; ratchanon.inpan@gmail.com (R.I.); jirarat.ka@cmu.ac.th (J.K.); pornsiri.p@cmu.ac.th (P.P.); ariyaphong.w@cmu.ac.th (A.W.)

**Keywords:** *Gymnema inodorum* extract, phytosome, phytonutrient delivery, anti-inflammation, anti-insulin resistance, macrophages, adipocytes

## Abstract

*Gymnema inodorum* (GI) is a leafy green vegetable found in the northern region of Thailand. A GI leaf extract has been developed as a dietary supplement for metabolic diabetic control. However, the active compounds in the GI leaf extract are relatively nonpolar. This study aimed to develop phytosome formulations of the GI extract to improve the efficiencies of their phytonutrients in terms of anti-inflammatory and anti-insulin-resistant activities in macrophages and adipocytes, respectively. Our results showed that the phytosomes assisted the GI extract’s dispersion in an aqueous solution. The GI phytocompounds were assembled into a phospholipid bilayer membrane as spherical nanoparticles about 160–180 nm in diameter. The structure of the phytosomes allowed phenolic acids, flavonoids and triterpene derivatives to be embedded in the phospholipid membrane. The existence of GI phytochemicals in phytosomes significantly changed the particle’s surface charge from neutral to negative within the range of −35 mV to −45 mV. The phytosome delivery system significantly exhibited the anti-inflammatory activity of the GI extract, indicated by the lower production of nitric oxide from inflamed macrophages compared to the non-encapsulated extract. However, the phospholipid component of phytosomes slightly interfered with the anti-insulin-resistant effects of the GI extract by decreasing the glucose uptake activity and increasing the lipid degradation of adipocytes. Altogether, the nano-phytosome is a potent carrier for transporting GI phytochemicals to prevent an early stage of T2DM.

## 1. Introduction

About 90% of diabetes cases are type 2 diabetes mellitus (T2DM), and the incidence of T2DM will continue to increase in the coming years [1]. The main cause of T2DM is obesity, which results from an increase in energy intake and the accumulation of lipids in adipocytes [2]. A high energy intake is related to an increase in pro-inflammatory cytokines (e.g., TNF-α, IL-1β and IL-6) that are produced by the hypothalamus [3]. These cytokines inhibit insulin signaling, resulting in dysfunction and insulin resistance in the adipocyte [4]. Insulin resistance further induces an increase in insulin release from a beta cell in order to keep the blood sugar level within a normal range. Later, the beta cell deteriorates and is unable to secrete insulin for maintaining glucose homeostasis. Finally, hyperglycemia occurs in the long term, and T2DM then develops [5]. 

Dark green and deep orange/yellow vegetables and non-starchy vegetables (NSVs) are recommended for dietary consumption with respect to adiposity and metabolic health. These vegetables contain micronutrients (e.g., vitamins, phenolic compounds, and carotenoids) and fibers that have protective properties against diabetic and related-metabolic syndromes [6,7]. Adequate vegetable consumption showed significant health-protective effects in obese humans. Cook et al. [8] found that overweight Latino youth between the ages of 8 and 18 years who consumed a greater quantity of vegetables showing a reduction in liver fat and visceral adipose tissue and improvement in insulin sensitivity. Moreover, Imai et al. [9] indicated that consuming vegetables before carbohydrates also helped slow the postprandial blood glucose effect. 

This study focuses on *Gymnema inodorum (Lour.)* Decne. It is one of the green leafy vegetables that is normally used in northern Thai cuisine. *G. inodorum* (GI) leaves have also been used as a folk medicine for diabetic treatments [10,11]. The GI leaf contains several groups of phytochemicals, such as phenolic acids, flavonoids, triterpenoid compounds, and pregnane glycosides [12,13,14,15]. GI phytonutrients can be extracted using water and alcoholic macerations or related techniques [16,17]. The GI extract (GIE) can lower blood glucose levels by preventing the intestinal absorption of glucose [16,18], reducing α-glucosidase and α-amylase activities in the digestive system [14]. Moreover, GIE exhibits antioxidant [11,19], anti-inflammatory [11] and insulin-mimetic activities [13,15]. However, the anti-diabetic compounds in GIE are relatively nonpolar [12], resulting in difficulties during membrane transport and a decrease in biological effects [20]. 

To date, encapsulation technology has been extensively used in the pharmaceutical and food industries. The encapsulation of bioactive compounds in the form of micro-sized and nanoscale colloidal particles depends on chemical ingredients and the methodology used to fabricate them [21]. The particles can increase absorption efficiency, specificity and the targeting ability of therapeutic agents [22,23]. In addition, therapeutic substances are protected from early degradation [24], they can enhance bioavailability, and their presence is prolonged in blood and cellular uptake [25]. When compared to a microparticle, a nanoparticle’s size remains homogeneous and exhibits a size between 10 and 500 nm [26]. For oral administration, nanoparticles at sizes lower than 200 nm show direct diffusion via the respiratory mucus layer [27] and intestinal mucosal sites [28]. 

The phytosome is one of the most powerful delivery systems for improving bioavailability and the health benefits of common plant constituents. The term “phyto” means plant, while “some” means cell-like [29]. The phytosome is a liposome-like structure made from phospholipids constructing a bilayer membrane. The phytoconstituents can be embedded in the membrane by forming H-bonds at the head group of phospholipids [30,31]. Many phytonutrients can be enclosed in the phytosome, including *Centella asiatica* extract [32], boswellic acids [33], quercetin [34], gymnemic acids [35], silymarin flavonolignans, curcuminoid polyphenols, green tea flavan-3-ol catechins, and grape seed catechin [36]. The phytosome can also be complexed into hyaluronic acid and gelling agents to increase the targeting ability of particles [37,38]. The consumption of the phytosome containing such a poorly absorbed phytoconstituent as a dietary supplement showed promising results and a significant increase in the bioavailability and biofunctionality of phytoconstituents when compared to the non-encapsulated phytosome [39,40]. 

This recent study aimed to study the effects of phytosome formulations containing GIE on anti-inflammation in macrophages and insulin-mimetic activity in adipocytes. The phytosomes were characterized in terms of size, surface charge and particle morphology. The encapsulation and loading efficiencies of GIE in phytosomes were calculated relative to the phytochemicals in the GIE before and after formulation. The phytochemicals included total phenolic acids, total flavonoids, total triterpenoids and the amount of (3*β*,16*β*)-16,28-dihydroxyolean-12-en-3-yl-*O*-*β*-D-glucopyranosyl-*β*-D-glucopyranosiduronic acid (GIA1). Anti-inflammation in macrophages was monitored by a reduction in nitric oxide production, while insulin resistance was measured by glucose uptake activity and lipolysis in adipocytes.

## 2. Materials and Methods

### 2.1. Materials

The leaves of *Gymnema inodorum* were collected from Fang District, Chiang Mai Province, Thailand. The plant specimen (CMUB herbarium voucher 38430) was authenticated and preserved at the Chiang Mai University Herbarium and Flora Database, Department of Biology, Faculty of Science, Chiang Mai University. All the analytical grade chemicals and solvents used in this study were purchased from Merck Ltd., Klongtoey, Thailand.

### 2.2. Preparation of G. inodorum Extract (GIE)

*G. inodorum* leaf samples were rinsed with clean water and dried in a hot-air oven at 70 °C for 24 h. The dry leaves were ground and soaked in 95% v/v ethanol with agitation. The extracting solvent was changed every 24 h for five days. Next, the extract solution was collected by filtration through a paper filter. After that, ethanol was removed with a rotary evaporator at 45 °C. The solution was then freeze-dried to obtain the ethanolic extract.

### 2.3. Preparation of Phytosome Nanoparticles

Phytosome formulations were prepared with the thin-film hydration method. The weight ratios of phosphatidylcholine (PC) and GIE were divided into three groups: (1) blank phytosome (B-phyto) containing PC:GIE at 1:0; (2) GIE-phytosome 1 (GIE-phyto1) containing PC:GIE at 1:1; and (3) GIE-phytosome 2 (GIE-phyto2) containing PC:GIE at 1:2. Briefly, the total ingredients (77 mg) were mixed and dissolved in 10 mL of ethanol in a round-bottomed flask. Next, the solvent was slowly removed with a rotary evaporator at 45 °C and 80 rpm under low pressure until a thin film was acquired. The thin film was rehydrated with 10 mL of phosphate-buffered saline at pH 7.4. The suspensions of phytosomes were carried out with an Avanti^®^ Mini-Extruder (Avanti Polar Lipid Inc., Alabaster, AL, USA), which was equipped with a polycarbonate membrane (0.1 µm pore size). Stock phytosome solutions (7.7 mg/mL) were kept at 4 °C for physicochemical characterizations and biological activity studies. The stability test of the phytosomes was observed during the experimental period (56 days). 

### 2.4. Physicochemical Characterizations of Phytosomal Nanoparticles

#### 2.4.1. Morphological Characterizations

Phytosome suspensions were diluted with 100× deionized water. Subsequently, each solution sample (20 µL) was dropped on a silicon wafer and dried for 4 h in a desiccator. Particle morphology was observed with Nano Search Microscopes LEXT OLS4500 (Olympus, Tokyo, Japan).

#### 2.4.2. Analysis of Particle Size, Polydispersity Index (PDI) and Zeta Potential of Phytosomal Nanoparticles

Phytosome samples were diluted 100 times with deionized water before measuring the particle size, PDI and zeta potential with a Zetasizer (Nano ZS, Malvern Instruments Ltd., Worcestershire, UK). 

#### 2.4.3. Encapsulation and Loading Efficiency of Nano-Phytosomes 

The entrapment efficiency and the loading efficiency of GIE in phytosomes were determined by an indirect method. Briefly, the suspensions of phytosomes were centrifuged at 15,000 rpm at 4 °C for 1 h. The supernatant was collected and used to determine the total contents of phenolic acids, flavonoids and GiA-1 ((3 β,1 6 β) -1 6,2 8 -dihydroxyolean-1 2 -en-3 -yl-O-β-D-glucopyranosyl-β-D182 glucopyranosiduronic acid). The isolation of GiA-1 was carried out using the protocol of Srinuanchai et al., 2021. The percentages of entrapment efficiency (%EE) and loading efficiency (%LE) were calculated using Equations (1) and (2):

(1) $$\%\mathrm{EE}=\frac{\left(\mathrm{amount}\;\mathrm{of}\;P/F/G\;\mathrm{in}\;\mathrm{total}\;\mathrm{GIE}\;\mathrm{nano}\;-\;\mathrm{amount}\;\mathrm{of}\;P/F/G\;\mathrm{in}\;\mathrm{free}\;\mathrm{GIE}\right)}{\mathrm{initial}\;\mathrm{amount}\;\mathrm{of}\;P/F/G\;\mathrm{in}\;\mathrm{GIE}}\times 100$$

(2) $$\%\mathrm{LE}=\frac{\left(\mathrm{amount}\;\mathrm{of}\;P/F/G\;\mathrm{in}\;\mathrm{total}\;\mathrm{GIE}\;\mathrm{nano}\;-\;\mathrm{amount}\;\mathrm{of}\;P/F/G\;\mathrm{in}\;\mathrm{free}\;\mathrm{GIE}\right)}{\mathrm{amount}\;\mathrm{of}\;P/F/G\;\mathrm{in}\;\mathrm{GIE}}\times 100$$

where *P* denotes the total phenolic content, *F* denotes the total flavonoid content and *G* denotes the amount of GiA-1.

#### 2.4.4. Total Phenolic Contents 

Briefly, 20 µL amounts of the samples were mixed with 100 µL of 10% (v/v) Folin–Ciocalteu reagent and incubated in the dark at room temperature for 3 min. Next, 80 µL of 7.5% w/v sodium carbonate was added and the mixture was further incubated in the dark for 30 min. The color intensity of the solutions was measured as the absorbance at 765 nm using a spectrophotometer (VICTOR Nivo, PerkinElmer Inc., Shelton, CT, USA). Gallic acid solutions (0–50 µg/mL) were used as a reference standard curve. The total phenolic content (TPC) was presented as milligram gallic acid equivalents per one gram of extract (mg GAE/g extract). 

#### 2.4.5. Total Flavonoid Contents

The sample solution (25 µL) was mixed with 125 µL of distilled water and 7.5 µL of 5% w/v sodium nitrite at room temperature for 6 min. After that, 15 µL of 10% w/v aluminum chloride was added and the mixture was further incubated for 6 min. Then, 50 µL of 1 M sodium hydroxide and 27.5 µL of deionized water were added. The mixture was kept in the dark for 15 min. The absorbance of the sample solutions was then measured at 415 nm with a spectrophotometer. The calibration curve was prepared using a quercetin solution at concentrations of 0–25 µg/mL. The total flavonoid content (TFC) of the samples was measured using the unit of mg quercetin equivalents per one gram of extract (mg QE/g extract).

#### 2.4.6. High Performance Liquid Chromatography (HPLC)

The GiA-1 content in the samples was analyzed with HPLC using an Agilent LC1260 HPLC system (Agilent Technologies, Santa Clara, CA, USA) with a photodiode array UV detector. The chromatographic separation was performed following the protocol of Srinuanchai et al., 2021. Briefly, standard GiA-1 and sample solutions were prepared in methanol and filtered through a 0.2 µm filter before injection into the HPLC system. A mobile phase consisted of 1% formic acid in acetonitrile (A) and 1% formic acid in water (B). The mobile phase was set at 0.6 mL/min with a gradient elution of A and B from 95%B:5%A (29 min) to 5%B:95%A (11 min) and further reverting to 95%B:5%A (10 min). The total run time was 50 min. The injection volume was 20 µL. 

### 2.5. Biological Activities 

#### 2.5.1. Sample Preparation

GIE and GiA-1 stock solutions (200 mg/mL) were prepared in 100% dimethyl sulfoxide (DMSO) (Sigma-Aldrich, St. Louis, MO, USA). The stock solutions of GIE, GiA-1 and B-phyto were diluted with Dulbecco’s modified eagle medium (DMEM) to obtain 800 µg/mL working solutions. The stock solutions of GIE-phyto1 and GIE-phyto2 were diluted with DMEM to obtain 1600 µg/mL and 2400 µg/mL working solutions, respectively, which contained a GIE equivalent at 800 µg/mL. The working solutions were filtered through a 0.2 µm diameter sterile syringe filter (Whatman, Buckinghamshire, UK). Different concentrations of the samples were further prepared by two-fold dilutions in DMEM for cytotoxicity and biological assays.

#### 2.5.2. Cell Culture

A RAW 267.4 macrophage cell line was obtained from CLS, Eppel-heim, Germany. The RAW macrophages were grown in an ultra-low attachment culture dish containing high glucose Dulbecco’s modified Eagle medium (DMEM) with L-glutamine supplemented with 10% Fetal Bovine Serum (FBS) and 1% antibiotic solution (penicillin and streptomycin) under 5% CO_2_ at 37 °C. In contrast, pre-adipocytes (3T3-L1 cell line) were cultured in DMEM including 10% FBS and 1% antibiotics at 37 °C in 5% CO_2_. After 2 days, the pre-adipocytes were differentiated to mature adipocytes by culturing in DMEM containing 0.5 mM 3-isobutyl-1-metylxanthine (IBMX), 1 mM dexamethasone, 167 µM insulin and 10% FBS for 72 h, followed by a DMEM solution containing insulin without IBMX and dexamethasone for 72 h. The differentiation was completed by incubating the cells in DMEM containing 10% FBS for 7–14 days.

#### 2.5.3. Cytotoxicity Test

The cytotoxicity was determined using an SRB assay in 96-well plates. Macrophage cells were seeded 2.5 × 10^4^ cells/well and incubated for 24 h. The 3T3-L1 pre-adipocytes were plated 3000 cells/well and differentiated to mature adipocytes. Then, the cells were treated with 100 µL of various concentrations of GIE, GiA-1, B-phyto, GIE-phyto1 and GIE-phyto2 samples at concentrations within the range of 25–800 µg/mL. Control cells in each treatment were incubated with DMEM containing an equivalent amount of DMSO or PBS. After 24 h of incubation, a 50% TCA solution was added for fixed cells at 4 °C for 1 h. After that, cells were removed from the solution and washed with water. The cells were air dried at room temperature overnight and stained with 0.057% SRB solution at room temperature for 30 min. After that, the cells were washed with 1% acetic acid and dried overnight. Finally, the cells were dissolved with 10 mM Tris-base pH 7.4. Optical density (OD) was measured at 510 nm using a microplate reader. A percentage of cellular viability can be calculated following Equation (3).

(3) $$\%\mathrm{Cell}\;\mathrm{viability}\;=\frac{\left(\mathrm{OD}\;\mathrm{of}\;\mathrm{treatment}\;-\;\mathrm{OD}\;\mathrm{of}\;\mathrm{blank}\right)}{\left(\mathrm{OD}\;\mathrm{of}\;\mathrm{control}\;-\;\mathrm{OD}\;\mathrm{of}\;\mathrm{blank}\right)}\times 100$$

#### 2.5.4. Anti-Inflammatory Activity

Anti-inflammatory activity was investigated using nitric oxide (NO) production from inflamed RAW 264.7 macrophage cells. Cell inflammation was induced by lipopolysaccharide (LPS). Briefly, macrophage cells were cultured in a 96-well plate at 2.5 × 10^4^ cells/well overnight. The cells were then co-treated with LPS (1 µg/mL) with or without a non-toxic dose of samples for 24 h. DMSO at 0.1% was used as the vehicle control. The production of NO (including nitrite and oxidized products of NO) in the cell culture medium was determined using a Griess reagent (1% sulfonamide and 0.1% N-(1-naphthyl) ethylenediamine dihydrochloride in 2.5% H_3_PO_4_). A culture medium (100 µL) was added with 100 µL of Griess reagent in a 96-well plate to develop color. Optical density (OD) was measured by spectrophotometry at 540 nm. The amount of nitric oxide production was determined in comparison with a sodium nitrite standard curve.

#### 2.5.5. Anti-Insulin Resistance Assay

Glucose uptake assay: Mature adipocytes were divided into two groups. The normal control group was treated with 0.1% DMSO. LPS groups were incubated with LPS (1 µg/mL) and 0.1% DMSO or with LPS and the samples at a non-toxic dose for 24 h. Upon LPS incubation, insulin resistance in adipocytes was developed. In the first step, the cells were washed twice with phosphate-buffered saline (PBS). The cells were incubated with an incomplete DMEM at 37 °C for 3 h. After that, cells were washed with PBS, and 1 mg/mL of BSA was further added (containing 80 µM 2-NBDG (2- [N-(7-nitrobenz-2-oxa-1, 3-diazol-4-yl) amino]-2-deoxy-glucose) and 1 µM insulin). Later, the cells were continuously incubated at 37 °C for 15 min. The fluorescent intensity of 2-NBDG was measured with a microplate reader using an excitation wavelength at 485 nm and an emission wavelength at 520 nm.

Lipolysis assay: The glycerol released by adipocytes was measured by using an adipolysis assay kit (Sigma Aldrich, St. Louis, MO, USA). Briefly, 25 µL of the culture medium was mixed with 200 µL of Free Glycerol Reagent in a 96-well plate. The mixture was incubated for 15 min at room temperature until color developed. Optical density (OD) was measured using a microplate reader at 540 nm. The glycerol content was calculated using standard glycerol in Equation (4).

(4) $$\mathrm{Glycerol}\;\mathrm{content}\;=\frac{\left(\mathrm{OD}\;\mathrm{of}\;\mathrm{sample}\;-\;\mathrm{OD}\;\mathrm{of}\;\mathrm{blank}\right)}{\left(\mathrm{OD}\;\mathrm{of}\;\mathrm{standard}\;-\;\mathrm{OD}\;\mathrm{of}\;\mathrm{blank}\right)}\times \mathrm{concentration}\;\mathrm{of}\;\mathrm{standard}$$

### 2.6. Statistical Analysis

Experimental data are expressed as means ± S.D. Statistical evaluation was carried out using one-way analysis of variance (one-way ANOVA) and Tukey’s post hoc test using GraphPad Prism (GraphPad Software, Inc., San Diego, CA, USA). Data groups with *p* values of less than 0.05 were considered statistically significant.

## 3. Results

### 3.1. Phytochemicals of G. inodorum Extract (GIE)

The GIE powder from the extraction process obtained about 19.04% w/w of dry leaf matter. The extract contained phenolics (24.79 ± 1.17 mg GAE/g extract), flavonoids (19.73 ± 0.65 mg QE/g extract) and 10.87 ± 0.03% w/w of GiA-1.

### 3.2. Characterizations of Phytosomes

**Figure 1:**
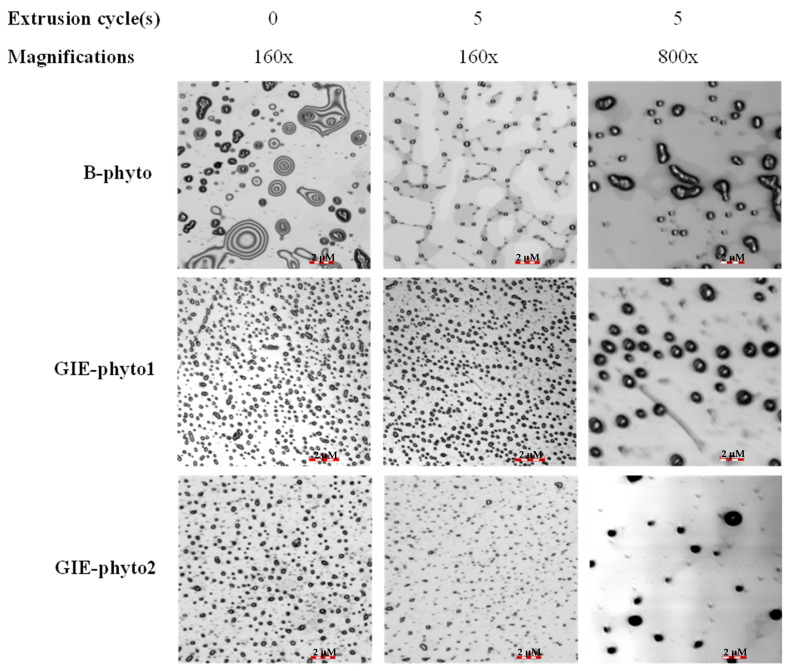
Particle morphology of phytosomes (B-phyto, GIE-phyto1 and GIE-phyto2) before and after extrusion, at magnifications of 160× with a scale bar of 10 µM and 800× with a scale bar of 2 µM.

**Figure 2:**
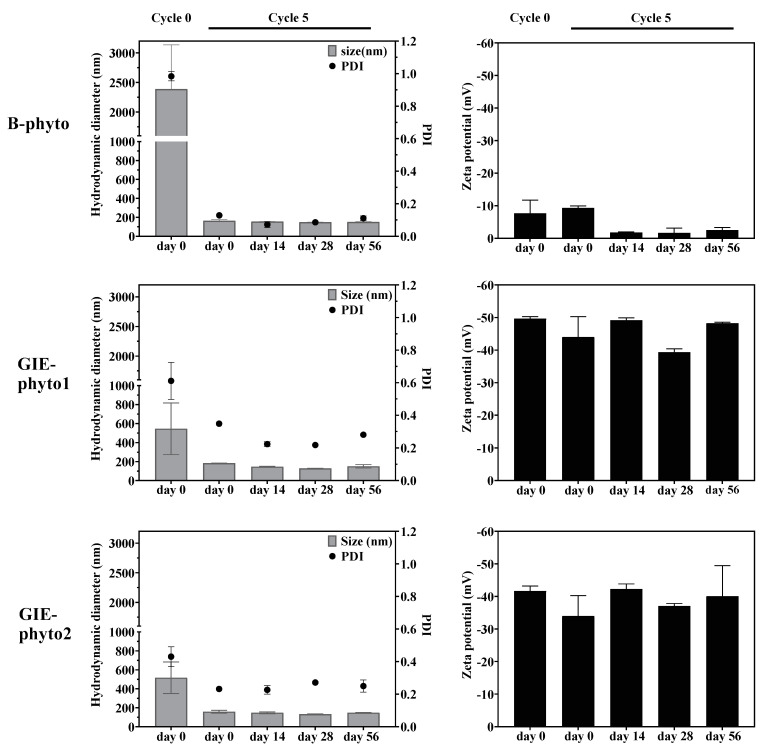
Hydrodynamic size, polydispersity index (PDI) and zeta potential of B-phyto, GIE-phyto1 and GIE-phyto2 before (cycle 0) and after (cycle 5) extrusion. The samples were observed from the production day (day 0) up until day 56.

In this study, phytosome formulations, such as B-phyto, GIE-phyto1 and GIE-phyto2, were constructed from different mass ratios of phosphatidylcholine (PC) and GIE. B-phyto contained pure PC, while GIE-phyto1 and GIE-phyto2 were mixtures of PC to GIE that were equivalent to 1:1 and 1:2, respectively. The phytosomes were enclosed by a phospholipid bilayer membrane, exhibiting a similarity to liposomes (Figure 1). The phytosomes were spherical and about 164 nm to 180 nm in diameter size. The nanoparticles’ sizes were homogeneously indicated by narrow polydispersity index (PDI) values that were less than 0.5. A difference in the particle surface charge of GIE-phyto1 and GIE-phyto2 was observed compared to B-phyto. The GIE loaded in GIE-phyto1 and GIE-phyto2 showed a highly negative charge, with a zeta potential ranging from −35 mV to −45 mV on their particle’s surface, while the B-phyto without GIE was relatively neutral. The phytosomes were stabilized at 4 °C for 56 days during the experimental periods. The particle size, PDI and zeta potential of phytosomes were not significantly different during storage. The results are shown in Figure 2. 

The phytosomes contained phenolic compounds, as observed by the total phenolic content and total flavonoid content and the amount of GiA-1 representing a triterpenoid marker in the GIE. The results are shown in Table 1. The phenolic compounds were preferably loaded in GIE-phyto1 and GIE-phyto2 in comparison with GiA-1, yet these two phytosome formulations had no differences with respect to the encapsulation and loading efficiencies of GIE.

### 3.3. Cytotoxicity of Samples in the RAW 264.7 Macrophage Cell Line and 3T3-L1 Adipocyte Cell Line 

The inhibitory concentrations of GIE, GiA-1, B-phyto, GIE-phyto1 and GIE-phyto2 on RAW 264.7 macrophages and 3T3-L1 adipocytes at 20% (IC_20_) and 50% (IC_50_) are shown in Table 2. In general, all treatments showed a poor cytotoxic effect on both cell lines, with IC_50_ values of more than 300 µg/mL. However, the IC_20_ and IC_50_ values suggested that RAW 264.7 macrophage cells were more sensitive to all treatments than 3T3-L1 adipose cells. Concentrations lower than IC_20_ were subsequently used in further experiments. 

### 3.4. Anti-Inflammatory Activity of Samples

**Figure 3:**
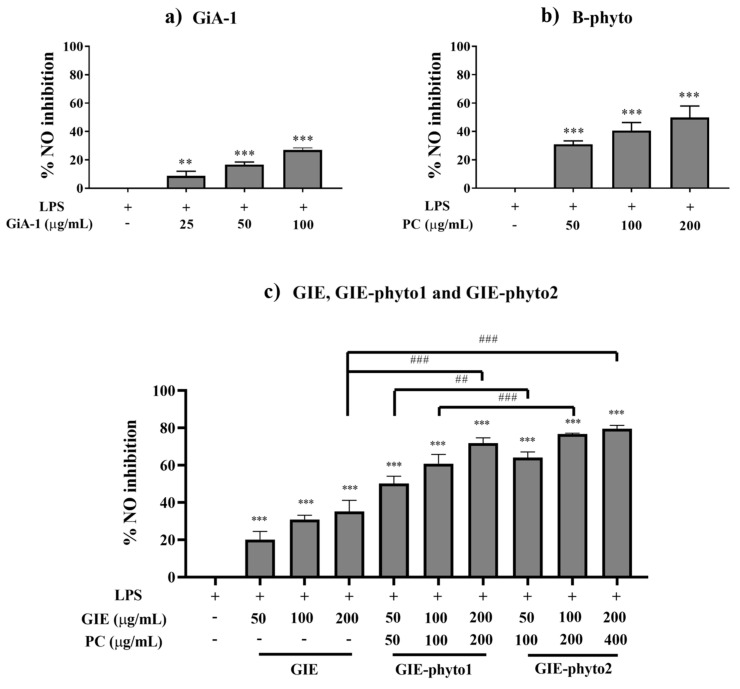
Inhibitory effects of (**a**) GiA–1, (**b**) B-phyto, (**c**) *G. inodorum* extract (GIE) and GIE in phytosome formulations (GIE-phyto1 and GIE-phyto2) on nitric oxide (NO) production from inflamed RAW 264.7 macrophage cells induced by lipopolysaccharide (LPS). The cells were treated with 1 µg/mL LPS and 0.1% DMSO or with 1 µg/mL LPS and the samples at various concentrations for 24 h. The percentages of NO inhibition are expressed relative to the control with the LPS treatment. ** and *** represent *p*-values of less than 0.01 and 0.001 compared to the LPS-treated control. ## and ### represent *p*-values of less than 0.01 and 0.001, compared in the groups of GIE, GIE-phyto1 and GIE-phyto2.

The results for anti-inflammatory activity by reductions in NO production revealed that GiA-1, GIE and each nanophytosome could significantly inhibit NO production in a dose-dependent manner (Figure 3). GiA-1, one of the bioactive compounds found in GIE, had NO inhibitory activity with percentage inhibitions of 8.70 ± 3.22%, 16.72 ± 1.70% and 26.96 ± 1.48% at 25, 50 and 100 μg/mL, respectively (Figure 3a). Furthermore, B-phyto at concentrations of 50, 100 and 200 µg/mL also revealed NO inhibition at 31 ± 2.38%, 41 ± 5.65% and 50 ± 8.07%, respectively (Figure 3b). As shown in Figure 3c, the inhibitory effects of the NO levels of GIE at all doses were lower than those of GIE-phyto1 and GIE-phyto2. The highest NO production inhibitory activity was observed in GIE-phyto2, followed by GIE-phyto1, B-phyto and GIE. 

### 3.5. Anti-Insulin-Resistance Activity of Samples

To investigate whether GIE, GiA-1 and GIE nanophytosomes exert an anti-insulin resistance effect on insulin-resistant adipocytes, 3T3-L1 adipocytes were incubated in the presence of 1 μg/mL LPS in combination with various concentrations of GIE, GiA-1, B-phyto, GIE-phyto1 and GIE-phyto2. Adipose cells and a conditioned medium were then analyzed with a 2-NBDG glucose uptake assay and lipolysis assay.

#### 3.5.1. Glucose Uptake

**Figure 4:**
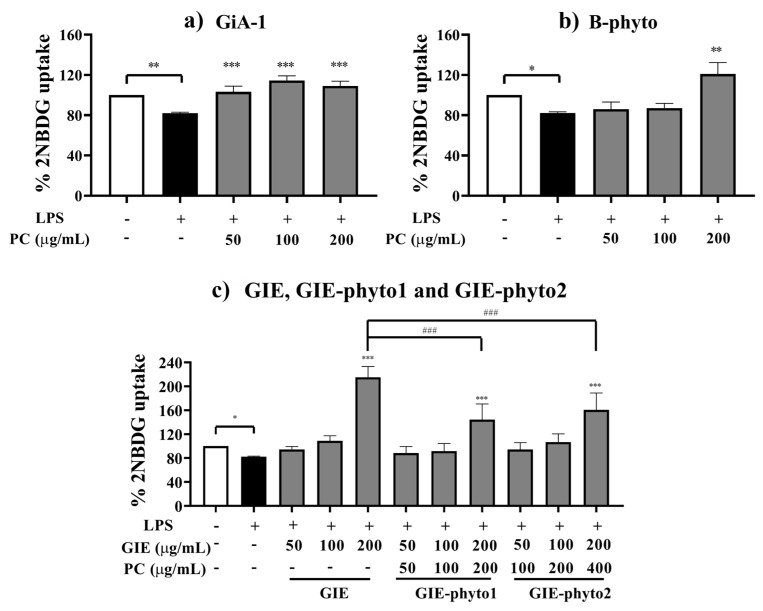
Effect of (**a**) GiA-1, (**b**) B-phyto, (**c**) *G. inodorum* extract (GIE) and GIE in phytosome formulations (GIE-phyto1 and GIE-phyto2) on LPS-induced insulin resistance with respect to 3T3-L1 adipocytes. The normal control group was treated with 0.1% DMSO. The LPS groups were treated with LPS (1 µg/mL) and 0.1% DMSO or with LPS and the samples at a non-toxic dose for 24 h. The percentages of 2-NBDG uptake are expressed relative to the control without LPS treatment. *, ** and *** represent *p*-values of less than 0.05, 0.01 and 0.001, compared to the LPS-treated control. ### represents *p*-values of less than 0.01 and 0.001, compared in the groups of GIE, GIE-phyto1 and GIE-phyto2.

Insulin resistance was induced in 3T3-L1 adipocytes by 1 μg/mL LPS. As shown in Figure 4, the LPS treatment caused a significant reduction in insulin-stimulated glucose uptake by approximately 20% when compared to the normal control group without LPS. Compared to LPS-treated adipocytes, insulin stimulating the 2-NBDG glucose uptake of cells treated with GiA-1 at 50 to 200 µg/mL (Figure 4a) and B-phyto at 200 μg/mL (Figure 4b) was significantly restored, and the restoration was close to the levels observed in the normal control group without LPS. Furthermore, all treatments at the concentration of 200 μg/mL, including GIE, GIE-phyto1 and GIE-phyto2, significantly improved the LPS-induced impairment of insulin-stimulated glucose uptake in adipocytes. Interestingly, the insulin-stimulated glucose uptake of insulin-resistant adipocytes treated with GIE-containing samples was 25–70% higher than that of insulin-sensitive adipocyte, a normal control group without LPS (Figure 4c). The GIE showed the highest anti-insulin-resistance activity, followed by GIE-phyto1 and GIE-phyto2.

#### 3.5.2. Lipolysis

**Figure 5:**
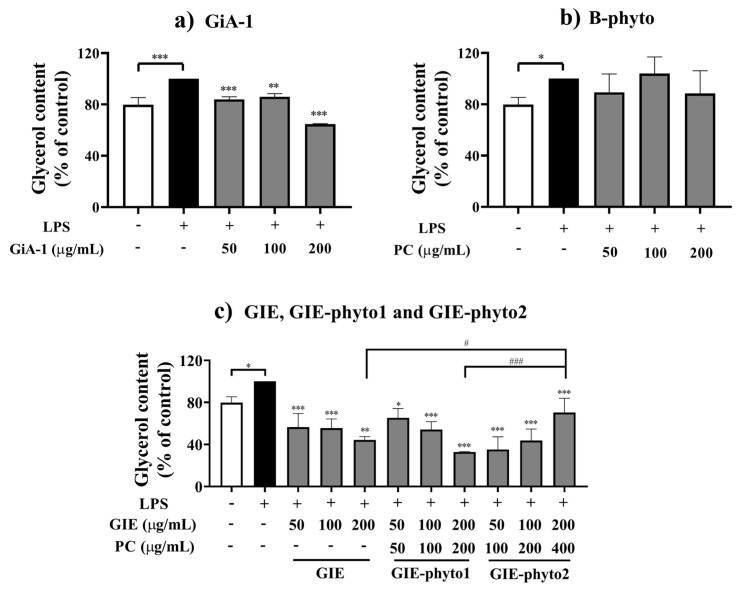
Effect of (**a**) GiA-1, (**b**) B-phyto, (**c**) *G. inodorum* extract (GIE) and GIE in phytosome formulations (GIE-phyto1 and GIE-phyto2) on glycerol release from LPS-induced insulin resistance on 3T3-L1 adipocytes. The normal control group was treated with 0.1% DMSO. The LPS groups were treated with LPS (1 µg/mL) and 0.1% DMSO or with LPS and the samples at a non-toxic dose for 24 h. The percentages of glycerol release are expressed relative to the control with LPS treatment. *, ** and *** represent *p*-values of less than 0.05, 0.01 and 0.001, compared to the LPS-treated control. # and ### represent *p*-values of less than 0.01 and 0.001, compared to the groups of GIE, GIE-phyto1 and GIE-phyto2.

The impairment of insulin sensitivity increased lipolysis in adipocytes. The rate of lipolysis was significantly induced by LPS, as determined by measuring the release of glycerol in the conditioned medium. As shown in Figure 5, 3T3-L1 adipocytes incubated for 24 h with LPS showed a 20% increase in glycerol release compared to a control group without LPS, demonstrating insulin-resistance in experimental cells. The treatment with GiA-1 (25–100 µg/mL) resulted in a 14–35% decrease in LPS-induced lipolysis, indicating an improvement in insulin sensitivity (Figure 5a). Interestingly, B-phyto at 50–200 µg/mL did not improve insulin sensitivity to inhibit LPS-induced adipocyte glycerol release (Figure 5b). As shown in Figure 5c, GIE at 50–200 µg/mL reduced LPS-induced insulin resistance with a 41–56% reduction in lipolysis. GIE-phyto1 at concentrations of 50–200 µg/mL also showed an anti-lipolytic effect, reducing glycerol release by 35–67%. Additionally, 50–200 µg/mL of GIE-phyto2 significantly improved insulin sensitivity by inhibiting 37–65% of LPS-induced glycerol release in adipocytes. GIE-phyto1 significantly improved LPS-induced insulin resistance in 3T3-L1 adipocytes in a dose-dependent manner. However, no significant differences were found in the antilipolytic activity observed between GIE-phyto1 and GIE treatments. In the case of GIE-phyto2, there was no significant difference in anti-lipolytic activity observed at 50 and 100 µg/mL compared to either GIE or GIE-phyto1. Interestingly, anti-lipolytic activity at a concentration of 200 µg/mL of GIE-phyto2 was observed significantly less than those observed in GIE and GIE-phyto1 treatments.

## 4. Discussion

*G. inodorum* (GI) is an edible plant exhibiting anti-diabetic benefits: hyperglycemic control and anti-inflammatory, antioxidant and anti-obesity effects [11,17]. Our study also suggested that the *G. inodorum* extract (GIE) prepared with 95% v/v ethanol significantly reduced the macrophage inflammation induced by lipopolysaccharide (LPS). The GIE inhibited the generation of nitric oxide (NO) in a dose-dependent manner, which is similar to the results reported by Dunkhunthod et al. [11]. It is possible that GIE can alleviate cell inflammation via similar mechanisms when RAW264.7 macrophage cells are treated with LPS and interferon-γ. GIE may help to decrease proinflammatory cytokine interleukin-6 and also downregulate the expression of cyclooxygenase-2 (COX-2), inducible nitric oxide synthase (iNOS) and IL-6 mRNA levels [11].

The LPS can promote the inflammation of adipocytes via Toll-like receptor 4, resulting in the dysfunction of glucose transport into adipocytes via insulin-dependent glucose transporter type 4 (GLUT4), which mimics insulin resistance in human obesity [41]. GLUT4 is damaged, leading to a decrease in glucose uptake levels in lipid cells [42]. In our study, the GIE improved the insulin sensitivity of the LPS-induced adipocyte model. After LPS-induced adipocytes were treated with the GIE, the cells significantly exhibited glucose transport into the cells. A lower dose of the GIE (≤100 µg/mL) seems to present anti-insulin-resistance activity since the glucose uptake level of inflamed adipocytes increased to an equivalent level of the insulin-treated group. Interestingly, a high dose of the GIE (up to 200 µg/mL) significantly improved insulin sensitivity in LPS-treated cells, leading to increased glucose uptake and decreased lipolysis. The treatment of insulin resistant 3T3-L1 adipocytes with GIE ranging from 50 to 200 µg/mL effectively stimulated glucose uptake in adipocytes by 112.5–275%. Importantly, GIE had higher effects on glucose uptake than the first-line anti-diabetic drug metformin. Previously, Qiu et al. [43] reported that metformin (129.2–1292 µg/mL) led to a much smaller increase in glucose uptake (by 133.3–160%) in PID1-induced insulin resistance in the 3T3-L1 adipocyte model. This report confirms a previous study by Qin et al. [44], who found that metformin (129.2–1292 µg/mL) also caused a smaller increase of 142.8–157.1% in glucose uptake in LYRM1-induced insulin resistance in the 3T3-L1 adipocyte model. However, the observed stimulation of the glucose uptake of GIE was much lower than the effect of rosiglitazone, an insulin-sensitizer and anti-diabetic drug in the class of thiazolidinedione. As reported by Zhang et al. [45], rosiglitazone (1.8–8.9 µg/mL) caused the stimulation of glucose uptake by 266.7–333.3% in high-glucose-induced insulin-resistant 3T3-L1 adipocytes.

In this study, the major component of triterpene saponin, (3*β*,16*β*)-16,28-dihydroxyolean-12-en-3-yl-*O*-*β*-D-glucopyranosyl-*β*-D-glucopyranosiduronic acid (GiA-1), was isolated following the protocol of Srinuanchai et al. [18]. GiA-1 was about 10.84% w/w in the GIE. It has been evidenced that (3*β*,4*α*,16*β*,22*α*)-22-(*N*-methylanthraniloxy)-16,23,28-trihidoroxyolean-12-en-3-yl-3-*O*-*β*-D-glucopyranosyl-*β*-D-glucopyranosiduronic acid (GiA-5) and several new oleanane triterpenoids isolated from *G. inodorum* leaves showed their insulin-mimetic activity by stimulating glucose uptake in 3T3-L1 adipocytes [15]. Therefore, our results suggested that GiA-1 is a promising compound with the ability to control intestinal glucose uptake by inhibiting α-glucosidase and sodium-glucose co-transporter type 1. Furthermore, we found that GiA-1 significantly inhibited NO production in LPS-induced macrophages in a dose-dependent manner. The GiA-1 compound also improved insulin sensitivity in LPS-treated cells, leading to an increase in glucose uptake and a decrease in lipolysis. Similar to the anti-inflammatory study, GiA-1 exhibited lower activities than GIE. These results also confirmed the possibility that GIE might contain other active compounds. 

The anti-inflammatory, anti-insulin-resistant and insulin-mimetic activities of the GIE could be related to other phytochemicals. Based on our preliminary results (see Supplementary Materials), GIE was fractionated following a low- to high-solvent polarity index by using hexane (Hex), dichloromethane (DCM), ethyl acetate (EtOAc) and methanol (MeOH). The fractions of Hex, DCM and EtOAc showed significantly higher anti-inflammatory abilities than the MeOH fraction. Furthermore, the phytochemicals of the fractions were investigated by gas chromatography mass spectrometry (GC/MS). The compounds distributed in Hex, DCM and EtOAc fractions exhibiting an anti-inflammatory potential were phytol [46], stigmasterol [47] and *α*- and *γ*-tocopherol (D and L isomers) [48]. Fewer compounds were observed in the MeOH fraction with the GC/MS technique. However, GiA-1 was observed only in the MeOH fraction (using HPLC analysis). The MeOH extract contained a high amount of GiA-1 (about 14.95% w/w), but it showed fewer lower anti-inflammatory effects as compared to non-polar fractions from Hex, DCM and EtOAc.

The non-polar fractions of Hex and EtOAc had greater insulin sensitivity activity than those of the DCM and MeOH extracts. Glucose molecules were remarkably transported into the inflamed adipocytes, and the effect of extracts was concentration-dependent. These results indicate that GiA-1 does not represent the active compound due to its absence in the Hex and EtOAc fractions. Unfortunately, the candidate compounds for anti-insulin-resistance and insulin-mimetic activities in the Hex and EtOAc fractions have not been detected by GC/MS. In the case of the MeOH fraction, a slight increase in the insulin sensitivity of LPS-induced adipocytes was also found in a dose-dependent manner. The active compounds may come partly from GiA-1 and other triterpene saponins [15]. Although GiA-1 is not a major active compound that exhibits anti-inflammatory and anti-insulin-resistant effects, GiA-1 can be used together with total phenolics and total flavonoids for indicating the encapsulation and loading efficiencies of the GIE extract in nano-phytosome formulations. 

The low water solubility of GIE could lead to low bioavailability, which is a general drawback that is similar to that for natural extracts that are prepared by organic solvents [49,50]. In our experiment, GIE had to be reconstituted in dimethyl sulfoxide (DMSO) for observing anti-inflammatory and anti-insulin-resistant activities in in vitro cell models. To improve the water solubility of the extract, the phytosome delivery system was chosen. The phytosome is similar to a liposome’s structure, which comprises phospholipid bilayers when dispersed in an aqueous solution [28,31]. For encapsulating natural compounds, phospholipids are dissolved together with a natural extract using an aprotic solvent, such as acetone, ethyl acetate or dioxane [32]. The phytocomponents are then bound to phospholipids by H-bonds and polar interactions [51]. After a solvent is removed, the mixture of the phospholipid–natural extract is reconstituted in an aqueous solution. Then, a lipid bilayer structure comprising a mixture of phospholipids and natural components is formed and ready for usage. Phytosomal products are commercially available on the food supplement market, for example Quercefit™ Phytosome, Ginkgoselect^®^ Phytosome, Ginseng Phytosome and Greenselect^®^/Green Tea Phytosome [31]. Riva et al. [34] indicated that phytosomes are highly absorbed in the GI tract. The dietary supplements of quercetin phytosome and quercetin were administrated orally in humans. Quercetin levels were found about 20 times higher in a subject group that consumed the quercetin phytosome than in the group with the quercetin dosage.

In our recent experiment, we successfully developed two formulations of phytosomes. The phytosomes formed unilamellar lipid bilayer nanovesicles with a homogeneity size of about 160–180 nm, as shown by optical microscope and dynamic light scattering. The phytosomes were highly dispersed in a buffer solution, and they were stable at 4 °C during our experimental period. The phytosomes containing GIE had a highly negative charge (about −35 mV to −60 mV) on the particle’s surface when compared to the blank phytosome (less than −10 mV). This could imply that the phytochemicals of the GIE are inserted in the phospholipid bilayer. The entrapment of phytochemicals could be the mixture of triterpene saponin GiA-1, phenolic acids and flavonoids. However, highly water-soluble phytocompounds may be entrapped inside core particles during nanoparticle formation by extrusion. The characteristics of the two phytosome formulas do not appear to be significantly different in terms of size, zeta potential, particle morphology, encapsulation, loading efficiencies and short-term stability.

The phytosome delivery system affected the anti-inflammatory and insulin-sensitizing actions of the GIE on in vitro cell models. The phytosomes with GIE significantly increased the anti-inflammatory activity of the extract twofold, inhibiting the pro-inflammatory NO mediator produced by the LPS-induced macrophage. The additive effect was a result of the anti-inflammatory effect of the phospholipid ingredient used to make phytosomes. A similar result was observed in previous studies by Park et al. [32] and Chiong et al. [52]. The inhibition of NO generation still occurred in a dose-dependent manner. A slight increase in the anti-inflammatory activity of GIE-phyto2 was observed. The effective inhibition of the generation of the NO mediator occurred in LPS-induced macrophage cells due to blank phytosomes and phytosomes with GIE. This could be due to the delivery of active components through the cell membrane and the engulfing ability of macrophage cells [36]. The typical characteristic of phytosomes is similar to liposomes due to the fusion of the lipid layer into a cell membrane. Then, the active ingredient entrapped in particles can be released in a cell [50]. 

Nonetheless, GIE-phytosomes decreased insulin sensitivity in LPS-induced adipocytes. The cells transported a lower amount of glucose and increased lipid degradation. The insulin-mimetic compounds in the GIE may be delivered into cells by the phytosome carrier rather than by affecting the embedding of GLUT4s on the cell membrane of adipocytes. In contrast, the phospholipid ingredient in phytosomes produced an adverse effect on the mechanism of lipid degradation in adipocytes. An increase in the amount of phospholipid ingredients in phytosome carriers tends to induce lipid degradation.

Taken together, our study proposes that GIE improves insulin sensitivity in an LPS-induced adipocyte model, investigating the use of GiA-1 content together with total phenolics and total flavonoids to indicate the encapsulation and loading efficiency of GIE extracts in nanophytosome formulations. Nonetheless, studies show that GiA-1 is not the main source of active compounds, and there are other nonpolar components of compounds with anti-insulin-resistance and insulin-mimetic activities in GIE.

## 5. Conclusions

GIE-nanophytosomes were successfully prepared using the thin-film hydration method. The nanoscale and monodisperse phytosome particles exhibited high entrapment efficiency and overcame the water insolubility of GIE. The anti-inflammatory activity of GIE nanophytosomes in LPS-stimulated RAW 264.7 macrophages was greater than that of the crude extract of *G. inodorum*. In addition, the anti-insulin-resistance abilities of GIE remained for GIE-nanophytosome formulations. Thus, the phytosome is a promising carrier for delivering the phytonutrients of the GI extract to enhance its anti-inflammatory activity and retain its anti-insulin-resistance ability. Furthermore, GiA-1, a major triterpenoid found in *G. inodorum*, exhibited fewer anti-inflammatory and anti-insulin-resistant activities than GIE. There may be other unknown active compositions in GIE that must be further elucidated. Moreover, the anti-inflammatory abilities, bioavailability, pharmaco-kinetics and safety of GIE-nanophytosomes in animals and humans should be evaluated to develop GIE-nanophytosomes as efficient candidates in pharmaceutical industries and the production of functional foods. 

## Figures and Tables

**Table 1:** Encapsulation and loading efficiencies (%EE and %LE) of the *G. inodorum* extract in phytosome formulations, based on the quantifications of total phenolic content (TPC), total flavonoid content (TFC) and GiA-1.

Samples	%EE			%LE		
	TPC	TFC	GiA-1	TPC	TFC	GiA-1
GIE-phyto1	17.49 ± 5.28	17.58 ± 5.62	41.06 ± 6.26	4.93 ± 1.55	14.17 ± 4.62	2.02 ± 0.31
GIE-phyto2	23.63 ± 7.92	11.96 ± 3.37	41.25 ± 4.47	5.53 ± 2.03	8.09 ± 2.57	1.39 ± 0.15

**Table 2:** The concentrations of GiA-1, B-phyto, GIE and GIE in phytosome formulations causing the cell death of the RAW267.3 macrophage and 3T3-L1 adipocyte at 20% (IC_20_) and 50% (IC_50_).

Samples	RAW267.3		3T3-L1	
	IC_20_ (µg/mL)	IC_50_ (µg/mL)	IC_20_ (µg/mL)	IC_50_ (µg/mL)
GiA-1	180.33 ± 15.11	379.20 ± 13.58	452.80 ± 59.59	688.80 ± 20.57
B-phyto	304.00 ± 45.25	>800.00	non-cytotoxicity	
GIE	492.80 ± 68.31	591.57 ± 57.05	424.00 ± 69.04	>800.00
GIE-phyto1	274.67 ± 75.21	635.20 ± 31.68	524.80 ± 90.51	>800.00
GIE-phyto2	222.67 ± 30.09	342.67 ± 34.65	441.07 ± 85.96	>800.00

## Data Availability

Data is contained within the article.

## Supplementary Materials

The following supporting information can be downloaded at: https://www.mdpi.com/article/10.3390/foods12112257/s1, Figure S1: Effects of Hex (A), DCM (B), EtOAc (C) and MeOH (D) on cell viability of RAW 264.7 macrophages and 3T3-L1 mature adipocytes; Figure S2: Inhibitory effects of the extracts prepared by hexane (Hex), dichloromethane (DCM), ethyl acetate (EtOAc) and methanol (MeOH) on nitric oxide (NO) production from inflamed RAW 264.7 macrophage cells induced by lipopolysaccharide (LPS); Figure S3: Effect of the extracts prepared by hexane (Hex), dichloromethane (DCM), ethyl acetate (EtOAc) and methanol (MeOH) on LPS- induced insulin resistance on 3T3-L1 adipocytes; Figure S4: Effect of the extracts prepared by hexane (Hex), dichloromethane (DCM), ethyl acetate (EtOAc) and methanol (MeOH) on glycerol release from LPS- induced insulin resistance on 3T3-L1 adipocytes; Table S1: Yield, total phenolic content (TPC) and total flavonoid content (TFC) in *G. inodorum* extracts prepared by organic solvents; Table S2: GC/MS phytochemicals profile of the *G. inodorum* extract prepared by hexane; Table S3: GC/MS phytochemicals profile of the *G. inodorum* extract prepared by dichloromethane; Table S4: GC/MS phytochemicals profile of the *G. inodorum* extract prepared by ethyl acetate; Table S5: GC/MS phytochemicals profile of the *G. inodorum* extract prepared by methanol. References [53,54,55,56,57,58,59,60,61,62,63,64,65,66,67,68,69,70,71,72,73,74,75,76,77,78,79,80,81,82,83,84,85,86,87,88,89,90,91,92,93,94,95] are cited in the supplementary materials.

## References

[1] Zheng Y., Ley S.H., Hu F.B. (2018). Global Aetiology and Epidemiology of Type 2 Diabetes Mellitus and Its Complications. Nat. Rev. Endocrinol..

[2] Romieu I., Dossus L., Barquera S., Blottière H.M., Franks P.W., Gunter M., Hwalla N., Hursting S.D., Leitzmann M., Margetts B. (2017). Energy Balance and Obesity: What are The Main Drivers?. CCC.

[3] De Souza C.T., Araujo E.P., Bordin S., Ashimine R., Zollner R.L., Boschero A.C., Saad M.J., Velloso L.A. (2005). Consumption of A Fat-Rich Diet Activates A Proinflammatory Response and Induces Insulin Resistance In The Hypothalamus. Endocrinology.

[4] Huang L.Y., Chiu C.J., Hsing C.H., Hsu Y.H. (2022). Interferon Family Cytokines in Obesity and Insulin Sensitivity. Cells.

[5] Cerf M.E. (2023). Beta Cell Dysfunction and Insulin Resistance. Front. Endocrinol..

[6] Pandey K.B., Rizvi S.I. (2009). Plant Polyphenols as Dietary Antioxidants in Human Health and Disease. Oxidative Med. Cell. Longev..

[7] Salas-Salvadó J., Becerra-Tomás N., Papandreou C., Bulló M. (2009). Dietary Patterns Emphasizing the Consumption of Plant Foods in the Management of Type 2 Diabetes: A Narrative Review. Adv. Nutr..

[8] Cook L.T., O’Reilly G.A., Goran M.I., Weigensberg M.J., Spruijt-Metz D., Davis J.N. (2014). Vegetable Consumption is Linked to Decreased Visceral and Liver Fat and Improved Insulin Resistance in Overweight Latino Youth. J. Acad. Nutr. Diet..

[9] Imai S., Kajiyama S., Kitta K., Miyawaki T., Matsumoto S., Ozasa N., Kajiyama S., Hashimoto Y., Fukui M. (2023). Eating Vegetables First Regardless of Eating Speed Has a Significant Reducing Effect on Postprandial Blood Glucose and Insulin in Young Healthy Women: Randomized Controlled Cross-Over Study. Nutrients.

[10] Panyadee P., Balslev H., Wangpakapattanawong P., Inta A. (2019). Medicinal Plants in Homegardens of Four Ethnic Groups in Thailand. J. Ethnopharmacol..

[11] Dunkhunthod B., Talabnin C., Murphy M., Thumanu K., Sittisart P., Eumkeb G. (2021). *Gymnema inodorum* (Lour.) Decne. Extract Alleviates Oxidative Stress and Inflammatory Mediators Produced by RAW264.7 Macrophages. Oxidative Med. Cell. Longev..

[12] Shimizu K., Ozeki M., Iino A., Nakajyo S., Urakawa N., Atsuchi M. (2001). Structure-Activity Relationships of Triterpenoid Derivatives Extracted from *Gymnema inodorum* Leaves on Glucose Absorption. Jpn. J. Pharmacol..

[13] Tiamyom K., Sirichaiwetchakoon K., Hengpratom T., Kupittayanant S., Srisawat R., Thaeomor A., Eumkeb G. (2019). The Effects of *Cordyceps sinensis* (Berk.) Sacc. and *Gymnema inodorum* (Lour.) Decne. Extracts on Adipogenesis and Lipase Activity In Vitro. Evid. Based Complement. Altern. Med..

[14] Trang D.T., Yen D.T.H., Cuong N.T., Anh L.T., Hoai N.T., Tai B.H., Doan V.V., Yen P.H., Quang T.H., Nhiem N.X. (2021). Pregnane Glycosides from *Gymnema inodorum* and Their α-Glucosidase Inhibitory Activity. Nat. Prod. Rep..

[15] An J.P., Park E.J., Ryu B., Lee B.W., Cho H.M., Doan T.P., Pham H.T.T., Oh W.K. (2020). Oleanane Triterpenoids from The Leaves of *Gymnema inodorum* and Their Insulin Mimetic Activities. J. Nat. Prod..

[16] Shimizu K., Ozeki M., Tanaka K., Itoh K., Nakajyo S., Urakawa N., Atsuchi M. (1997). Suppression of Glucose Absorption by Extracts from The Leaves of *Gymnema inodorum*. J. Vet. Med. Sci..

[17] Bespinyowong R., Pongthananikorn S., Chiabchalard A. (2013). Efficacy and Safety of *Gymnema inodorum* Tea Consumption in Type 2 Diabetic Patients. Chula. Med. J..

[18] Srinuanchai W., Nooin R., Pitchakarn P., Karinchai J., Suttisansanee U., Chansriniyom C., Jarussophon S., Temviriyanukul P., Nuchuchua O. (2021). Inhibitory Effects of *Gymnema inodorum* (Lour.) Decne Leaf Extracts and Its Triterpene Saponin on Carbohydrate Digestion and Intestinal Glucose Absorption. J. Ethnopharmacol..

[19] Jeytawan N., Yadoung S., Jeeno P., Yana P., Sutan K., Naksen W., Wongkaew M., Sommano S., Hongsibsong S. (2022). Antioxidant and Phytochemical Potential and Phytochemicals in *Gymnema inodorum* (Lour.) Decne in Northern Thailand. Plants.

[20] Martel F., Monteiro R., Calhau C. (2010). Effect of Polyphenols on The Intestinal and Placental Transport of Some Bioactive Compounds. Nutr. Res. Rev..

[21] McClements D.J. (2012). Nanoemulsions Versus Microemulsions: Terminology, Differences, and Similarities. Soft Matter.

[22] Li B., Wang F., Gui L., He Q., Yao Y., Chen H. (2018). The Potential of Biomimetic Nanoparticles for Tumor-Targeted Drug Delivery. Nanomedicine.

[23] Siles-Sánchez M.d.l.N., Jaime L., Villalva M., Santoyo S. (2022). Encapsulation of Marjoram Phenolic Compounds Using Chitosan to Improve Its Colon Delivery. Foods.

[24] Li Q., Wang L., Zheng M., Lu H., Liu Y., Wang Y., Lu S. (2023). Microencapsulation with Different Starch-Based Polymers for Improving Oxidative Stability of Cold-Pressed Hickory (*Carya cathayensis* Sarg.) Oil. Foods.

[25] Kumari A., Yadav S.K., Yadav S.C. (2010). Biodegradable Polymeric Nanoparticlesbased Drug Delivery Systems. Colloids Surf. B Biointerfaces.

[26] McClements D.J. (2018). Encapsulation, Protection, and Delivery of Bioactive Proteins and Peptides Using Nanoparticle and Microparticle Systems: A Review. Adv. Colloid Interface Sci..

[27] Schuster B.S., Suk J.S., Woodworth G.F., Hanes J. (2013). Nanoparticle Diffusion in Respiratory Mucus from Humans Without Lung Disease. Biomaterials.

[28] Primard C., Rochereau N., Luciani E., Genin C., Delair T., Paul S., Verrier B. (2010). Traffic of Poly(Lactic Acid) Nanoparticulate Vaccine Vehicle from Intestinal Mucus to Sub-Epithelial Immune Competent Cells. Biomaterials.

[29] Suryawanshi J.S. (2011). Phytosome: An Emerging Trend in Herbal Drug Treatment. JMGG.

[30] Ghanbarzadeh B., Babazadeh A., Hamishehkar H. (2016). Nano-Phytosome as A Potential Food-Grade Delivery System. Food Biosci..

[31] Barani M., Sangiovanni E., Angarano M., Rajizadeh M.A., Mehrabani M., Piazza S., Gangadharappa H.V., Pardakhty A., Mehrbani M., Dell’Agli M. (2021). Phytosomes as Innovative Delivery Systems for Phytochemicals: A Comprehensive Review of Literature. Int. J. Nanomed..

[32] Park J.H., Jang J.S., Kim K.C., Hong J.T. (2018). Anti-Inflammatory Effect of Centella Asiatica Phytosome in A Mouse Model of Phthalic Anhydride-Induced Atopic Dermatitis. Phytomedicine.

[33] Hüsch J., Bohnet J., Fricker G., Skarke C., Artaria C., Appendino G., Schubert-Zsilavecz M., Abdel-Tawab M. (2013). Enhanced Absorption of Boswellic Acids by A Lecithin Delivery form (Phytosome^®^) of Boswellia Extract. Fitoterapia.

[34] Riva A., Ronchi M., Petrangolini G., Bosisio S., Allegrini P. (2019). Improved Oral Absorption of Quercetin from Quercetin Phytosome^®^, A New Delivery System Based on Food Grade Lecithin. Eur. J. Drug Metab. Pharmacokinet..

[35] Pathan R.A., Bhandari U. (2011). Gymnemic Acid-Phospholipid Complex: Preparation and Characterization. J. Dispers. Sci. Technol..

[36] Kidd P.M. (2009). Bioavailability and Activity of Phytosome Complexes from Botanical Polyphenols: The Silymarin, Curcumin, Green Tea, and Grape Seed Extracts. Altern. Med. Rev..

[37] Abdelkader H., Longman M.R., Alany R.G., Pierscionek B. (2016). Phytosome-Hyaluronic Acid Systems for Ocular Delivery of L-Carnosine. Int. J. Nanomed..

[38] El-Menshawe S.F., Ali A.A., Rabeh M.A., Khalil N.M. (2018). Nanosized Soy Phytosome-Based Thermogel as Topical Anti-Obesity Formulation: An Approach for Acceptable Level of Evidence of An Effective Novel Herbal Weight Loss Product. Int. J. Nanomed..

[39] Chen Z.P., Sun J., Chen H.X., Xiao Y.Y., Liu D., Chen J., Cai H., Cai B.C. (2010). Comparative Pharmacokinetics and Bioavailability Studies of Quercetin, Kaempferol and Isorhamnetin After Oral Administration of *Ginkgo biloba* Extracts, *Ginkgo biloba* Extract Phospholipid Complexes and *Ginkgo biloba* Extract Solid Dispersions in Rats. Fitoterapia.

[40] Sahin O.I., Dundar A.N., Ozdemir S., Uzuner K., Parlak M.E., Dagdelen A.F., Saricaoglu F.T. (2022). Nanophytosomes as A Protection System to Improve the Gastrointestinal Stability and Bioavailability of Phycocyanin. Food Biosci..

[41] Jackson S.E., Beeken R.J., Wardle J. (2015). Obesity, Perceived Weight Discrimination, and Psychological Well-Being in Older Adults in England. Obesity.

[42] Chang L., Chiang S.H., Saltiel A.R. (2004). Insulin Signaling and The Regulation of Glucose Transport. Mol. Med..

[43] Qiu J., Wang Y.M., Shi C.M., Yue H.N., Qin Z.Y., Zhu G.Z., Cao X.G., Ji C.B., Cui Y., Guo X.R. (2012). NYGGF4 (PID1) Effects on Insulin Resistance are Reversed by Metformin in 3T3-L1 Adipocytes. J. Bioenerg. Biomembr..

[44] Qin Z.Y., Zhang M., Dai Y.M., Wang Y.M., Zhu G.Z., Zhao Y.P., Ji C.B., Qiu J., Cao X.G., Guo X.R. (2014). Metformin prevents LYRM1-induced insulin resistance in 3T3-L1 adipocytes via a mitochondrial-dependent mechanism. Exp. Biol. Med..

[45] Zhang W.Y., Lee J.J., Kim Y., Kim I.S., Han J.H., Lee S.G., Ahn M.J., Jung S.H., Myung C.S. (2012). Effect of eriodictyol on glucose uptake and insulin resistance in vitro. J. Agric. Food Chem..

[46] Carvalho A.M.S., Heimfarth L., Pereira E.W.M., Oliveira F.S., Menezes I.R.A., Coutinho H.D.M., Picot L., Antoniolli A.R., Quintans J.S.S., Quintans-Júnior L.J. (2020). Phytol, A Chlorophyll Component, Produces Antihyperalgesic, Anti-inflammatory, and Antiarthritic Effects: Possible NFκB Pathway Involvement and Reduced Levels of the Proinflammatory Cytokines TNF-α and IL-6. J. Nat. Prod..

[47] Morgan L.V., Petry F., Scatolin M., De Oliveira P.V., Alves B.O., Zilli G.A.L., Volfe C.R.B., Oltramari A.R., De Oliveira D., Scapinello J. (2021). Investigation of The Anti-Inflammatory Effects of Stigmasterol in Mice: Insight into Its Mechanism of Action. Behav. Pharmacol..

[48] Reiter E., Jiang Q., Christen S. (2007). Anti-Inflammatory Properties of Alpha- And Gamma-Tocopherol. Mol. Asp. Med..

[49] Chairuk P., Tubtimsri S., Jansakul C., Sriamornsak P., Weerapol Y. (2020). Enhancing Oral Absorption of Poorly Water-Soluble Herb (*Kaempferia parviflora*) Extract Using Self-Nanoemulsifying Formulation. Pharm. Dev. Technol..

[50] Boonyarattanasoonthorn T., Kijtawornrat A., Songvut P., Nuengchamnong N., Buranasudja V., Khemawoot P. (2022). Increase Water Solubility of *Centella asiatica* Extract by Indigenous Bioenhancers Could Improve Oral Bioavailability and Disposition Kinetics of Triterpenoid Glycosides in Beagle dogs. Sci. Rep..

[51] Hou Z., Li Y., Huang Y., Zhou C., Lin J., Wang Y., Cui F., Zhou S., Jia M., Ye S. (2013). Phytosomes Loaded with Mitomycin C-Soybean Phosphatidylcholine Complex Developed for Drug Delivery. Mol. Pharm..

[52] Chiong H.S., Yong Y.K., Ahmad Z., Sulaiman M.R., Zakaria Z.A., Yuen K.H., Hakim M.N. (2013). Cytoprotective and Enhanced Anti-Inflammatory Activities of Liposomal Piroxicam Formulation in Lipopolysaccharide-Stimulated RAW 264.7 Macrophages. Int. J. Nanomed..

[53] Ali A.M., Gabbar M.A., Abdel-Twab S.M., Fahmy E.M., Ebaid H., Alhazza I.M., Ahmed O.M. (2020). Antidiabetic Potency, Antioxidant Effects, and Mode of Actions of *Citrus reticulata* Fruit Peel Hydroethanolic Extract, Hesperidin, And Quercetin in Nicotinamide/Streptozotocin-Induced Wistar Diabetic Rats. Oxidative Med. Cell. Longev..

[54] Alonso-Castro A.J., Serrano-Vega R., Pérez Gutiérrez S., Isiordia-Espinoza M.A., Solorio-Alvarado C.R. (2022). Myristic Acid Reduces Skin Inflammation and Nociception. J. Food Biochem..

[55] Amor I.L.-B., Boubaker J., Sgaier M.B., Skandrani I., Bhouri W., Neffati A., Kilani S., Bouhlel I., Ghedira K., Chekir-Ghedira L. (2009). Phytochemistry and Biological Activities of Phlomis Species. J. Ethnopharmacol..

[56] Arai R., Nukazawa K., Kazama S., Takemon Y. (2015). Variation in Benthic Invertebrate Abundance Along Thermal Gradients Within Headwater Streams of a Temperate Basin in Japan. Hydrobiologia.

[57] Azemi A.K., Mokhtar S.S., Rasool A.H.G. (2021). *Clinacanthus nutans*: Its Potential Against Diabetic Vascular Diseases. Braz. J. Pharm. Sci..

[58] Dey P., Mah E., Li J., Jalili T., Symons J.D., Bruno R.S. (2018). Improved Hepatic γ-Tocopherol Status Limits Oxidative and Inflammatory Stress-Mediated Liver Injury in *db/db* Mice with Nonalcoholic Steatohepatitis. J. Funct. Foods.

[59] Djuricic I., Calder P.C. (2021). Beneficial Outcomes of Omega-6 and Omega-3 Polyunsaturated Fatty Acids on Human Health: An Update for 2021. Nutrients.

[60] Elmazar M.M., El-Abhar H.S., Schaalan M.F., Farag N.A. (2013). Phytol/Phytanic Acid and Insulin Resistance: Potential Role of Phytanic Acid Proven by Docking Simulation and Modulation of Biochemical Alterations. PLoS ONE.

[61] Ezirim C.Y., Abarikwu S.O., Uwakwe A.A., Mgbudom-Okah C.J. (2019). Protective Effects of *Anthocleista djalonensis* A. Chev Root Extracts Against Induced Testicular Inflammation and Impaired Spermatogenesis in Adult Rats. Mol. Biol. Rep..

[62] Fazelipour S., Hadipour Jahromy M., Tootian Z., Goodarzi N. (2021). Antidiabetic Effects of the Ethanolic Extract of *Allium saralicum* RM Fritsch on Streptozotocin-Induced Diabetes in A Mice Model. Food Sci. Nutr..

[63] Ferdous A., Janta R.A., Arpa R.N., Afroze M., Khan M., Moniruzzaman M. (2020). The Leaves of *Bougainvillea spectabilis* Suppressed Inflammation and Nociception In Vivo Through the Modulation of Glutamatergic, Cgmp, And ATP-Sensitive K+ Channel Pathways. J. Ethnopharmacol..

[64] Ganbold M., Ferdousi F., Arimura T., Tominaga K., Isoda H. (2020). New Amphiphilic Squalene Derivative Improves Metabolism of Adipocytes Differentiated from Diabetic Adipose-Derived Stem Cells and Prevents Excessive Lipogenesis. Front. Cell Dev. Biol..

[65] George L.O., Radha H.R., Somasekariah B.V. (2018). In Vitro Anti-Diabetic Activity and GC-MS Analysis of Bioactive Compounds Present in the Methanol Extract of *Kalanchoe pinnata*. NISCAIR-CSIR.

[66] Gonçalves N.B., Bannitz R.F., Silva B.R., Becari D.D., Poloni C., Gomes P.M., Foss M.C., Foss-Freitas M.C. (2018). A-Linolenic Acid Prevents Hepatic Steatosis and Improves Glucose Tolerance in Mice Fed a High-Fat Diet. Clinics.

[67] Ibrahim N., Naina Mohamed I. (2021). Interdependence of Anti-Inflammatory and Antioxidant Properties of Squalene–Implication for Cardiovascular Health. Life.

[68] Islam M.T., Ayatollahi S.A., Zihad S.N.K., Sifat N., Khan M.R., Paul A., Salehi B., Islam T., Mubarak M.S., Martins N. (2020). Phytol Anti-Inflammatory Activity: Pre-Clinical Assessment and Possible Mechanism of Action Elucidation. Cell. Mol. Biol..

[69] Khalil A.S.M., Giribabu N., Yelumalai S., Shahzad H., Kilari E.K., Salleh N. (2021). Myristic Acid Defends Against Testicular Oxidative Stress, Inflammation, Apoptosis: Restoration of Spermatogenesis, Steroidogenesis in Diabetic Rats. Life Sci..

[70] Kim D.Y., Kim J., Ham H.J., Choue R. (2013). Effects Of D-A-Tocopherol Supplements on Lipid Metabolism in A High-Fat Diet-Fed Animal Model. Nutr. Res. Pract..

[71] Li L., Wang Q., Yang Y., Wu G., Xue-Lei X., Aisa H. (2012). Chemical Components and Antidiabetic Activity of Essential Oils Obtained by Hydrodistillation and Three Solvent Extraction Methods from *Carthamus tinctorius* L.. Acta Chromatogr..

[72] López-Gómez C., Santiago-Fernández C., García-Serrano S., García-Escobar E., Gutiérrez-Repiso C., Rodríguez-Díaz C., Ho-Plágaro A., Martín-Reyes F., Garrido-Sánchez L., Valdés S. (2020). Oleic Acid Protects Against Insulin Resistance by Regulating the Genes Related to the PI3K Signaling Pathway. J. Clin. Med..

[73] Mahmood R., Kayani W.K., Ahmed T., Malik F., Hussain S., Ashfaq M., Ali H., Rubnawaz S., Green B.D., Calderwood D. (2020). Assessment of Antidiabetic Potential and Phytochemical Profiling of Rhazya Stricta Root Extracts. BMC Complement. Med. Ther..

[74] Matsuda H., Suzuki D., Asakura M., Ooi S., Saitoh R., Otokozawa R., Shirai T. (2018). Effects of Dietary Phytol on Glucose Uptake and Insulin Secretion In Vitro and In Vivo. Food Nutr. Current Res..

[75] Nasution R., Fitrah C.N., Helwati H., Murniana A.B., Cutchamzurni C. (2018). Antidiabetes Activities Extract Hexane from the Peels of *Artocarpus camansi* Blanco Fruit. Asian J. Pharm. Clin. Res..

[76] Okechukwu P.N. (2020). Evaluation of Anti-Inflammatory, Analgesic, Antipyretic Effect of Eicosane, Pentadecane, Octacosane, and Heneicosane. Asian J. Pharm. Clin. Res..

[77] Okokon J.E., Etuk I.C., Thomas P.S., Drijfhout F.P., Claridge T.D.W., Li W.-W. (2022). In Vivo Antihyperglycaemic and Antihyperlipidemic Activities and Chemical Constituents of *Solanum anomalum*. Biomed. Pharmacother. Biomedecine Pharmacother..

[78] Pang K.-L., Chin K.-Y. (2019). The Role of Tocotrienol in Protecting Against Metabolic Diseases. Molecules.

[79] Pauls S.D., Rodway L.A., Winter T., Taylor C.G., Zahradka P., Aukema H.M. (2018). Anti-Inflammatory Effects of α-Linolenic Acid in M1-Like Macrophages are Associated with Enhanced Production of Oxylipins From α-Linolenic and Linoleic Acid. J. Nutr. Biochem..

[80] Ratheesh M., Sunil S., Sheethal S., Jose S.P., Sandya S., Ghosh O.S.N., Rajan S., Jagmag T., Tilwani J. (2022). Anti-Inflammatory and Anti-COVID-19 Effect of a Novel Polyherbal Formulation (Imusil) Via Modulating Oxidative Stress, Inflammatory Mediators and Cytokine Storm. Inflammopharmacology.

[81] Roshankhah S., Abdolmaleki A., Salahshoor M.R. (2020). Anti-Inflammatory, Anti-Apoptotic, and Antioxidant Actions of Middle Eastern Phoenix Dactylifera Extract on Mercury-Induced Hepatotoxicity In Vivo. Mol. Biol. Rep..

[82] Saraswathi V., Kumar N., Ai W., Gopal T., Bhatt S., Harris E.N., Talmon G.A., Desouza C.V. (2022). Myristic Acid Supplementation Aggravates High Fat Diet-Induced Adipose Inflammation and Systemic Insulin Resistance in Mice. Biomolecules.

[83] Sulaimon L.A., Anise E.O., Obuotor E.M., Samuel T.A., Moshood A.I., Olajide M., Fatoke T. (2020). In Vitro Antidiabetic Potentials, Antioxidant Activities and Phytochemical Profile of African Black Pepper (*Piper guineense*). Clin. Phytoscience.

[84] Tham Y.Y., Choo Q.C., Muhammad T.S.T., Chew C.H. (2020). Lauric Acid Alleviates Insulin Resistance by Improving Mitochondrial Biogenesis in THP-1 Macrophages. Mol. Biol. Rep..

[85] Tian X., Seluanov A., Gorbunova V. (2017). Molecular Mechanisms Determining Lifespan in Short-And Long-Lived Species. Trends Endocrinol. Metab..

[86] Ujita M., Nagayama H., Kanie S., Koike S., Ikeyama Y., Ozaki T., Okumura H. (2009). Carbohydrate Binding Specificity of Recombinant Human Macrophage Β-Glucan Receptor Dectin-1. Biosci. Biotechnol. Biochem..

[87] Wang D.Q., Liu X.L., Rong Q.F., Han L., Zhao N.Q. (2013). Alpha-Linolenic Acid Improves Insulin Sensitivity in Obese Patients. Zhonghua Yi Xue Za Zhi.

[88] Wang J., Huang M., Yang J., Ma X., Zheng S., Deng S., Huang Y., Yang X., Zhao P. (2017). Anti-Diabetic Activity of Stigmasterol from Soybean Oil by Targeting Tte GLUT4 Glucose Transporter. Food Nutr. Res..

[89] Ward M.G., Li G., Barbosa-Lorenzi V.C., Hao M. (2017). Stigmasterol Prevents Glucolipotoxicity Induced Defects in Glucose-Stimulated Insulin Secretion. Sci. Rep..

[90] Xia M., Liu L., Qiu R., Li M., Huang W., Ren G., Zhang J. (2018). Anti-Inflammatory and Anxiolytic Activities of *Euphorbia hirta* Extract in Neonatal Asthmatic Rats. AMB Express.

[91] Yeh C.-F., Chuang T.-Y., Hung Y.-W., Lan M.-Y., Tsai C.-H., Huang H.-X., Lin Y.-Y. (2019). Soluble Epoxide Hydrolase Inhibition Enhances Anti-Inflammatory and Antioxidative Processes, Modulates Microglia Polarization, and Promotes Recovery After Ischemic Stroke. Neuropsychiatr. Dis. Treat.

[92] Yoon S.-Y., Ahn D., Hwang J.Y., Kang M.J., Chung S.J. (2021). Linoleic Acid Exerts Antidiabetic Effects by Inhibiting Protein Tyrosine Phosphatases Associated with Insulin Resistance. J. Funct. Foods.

[93] Zafar H., Mirza I.A., Hussain W., Fayyaz M. (2021). Comparative Efficacy of Tocotrienol and Tocopherol for Their Anti Diabetic Effects. Biomed. J. Sci. Tech. Res..

[94] Zaky A.S., Kandeil M., Abdel-Gabbar M., Fahmy E.M., Almehmadi M.M., Ali T.M., Ahmed O.M. (2022). The Antidiabetic Effects and Modes of Action of The *Balanites aegyptiaca* Fruit and Seed Aqueous Extracts in NA/STZ-Induced Diabetic Rats. Pharmaceutics.

[95] Zhong R.-F., Xu G.-B., Wang Z., Wang A.-M., Guan H.-Y., Li J., He X., Liu J.-H., Zhou M., Li Y.-J. (2015). Identification of Anti-Inflammatory Constituents from Kalimeris Indica with UHPLC-ESI-Q-TOF-MS/MS and GC–MS. J. Ethnopharmacol..

