# Supplementary material for: Phytosome Supplements for Delivering Gymnema inodorum Phytonutrients to Prevent Inflammation in Macrophages and Insulin Resistance in Adipocytes
Source: Foods. 2023 Jun 3;12(11):2257. doi: 10.3390/foods12112257 (PMC10253226; doi:10.3390/foods12112257)
Supplement: Supplementary file 1 [file foods-12-02257-s001.zip › foods-2382030-supplementary.pdf]

Phytosome supplements for delivering *Gymnema inodorum* phytonutrients to prevent inflammation in macrophages and insulin resistance in adipocytes

Onanong Nuchuchua[1,#], Ratchanon Inpan[2,#], Wanwisa Srinuanchai[1], Jirarat Karinchai[2], Pornsiri Pitchakarn[2], Ariyaphong Wongnoppavich [2], Arisa Imsumran[2,*]

[1]National Nanotechnology Center (NANOTEC), National Science and Technology Development Agency (NSTDA), Pathum Thani 12120, Thailand

[2]Department of Biochemistry, Faculty of Medicine, Chiang Mai University, Chiang Mai 50200, Thailand

[#] These authors contributed equally to this work.

* Corresponding author: Asst. Prof. Dr. Arisa Imsumran Bonness, Department of Biochemistry, Faculty of Medicine, Chiang Mai University, Chiang Mai

Tel.: +668 3 477 4151

E-mail address: arisa.bonness@cmu.ac.th

Table S1. Yield, total phenolic content (TPC) and total flavonoid content (TFC) in *G. inodorum* extracts prepared by organic solvents from low to high polarity index, such as hexane (HEX), dichloromethane (DCM), ethyl acetate (EtOAc) and methanol (MeOH).

**Extracts**	**Yield (%w/w)**	**TPC (mg GAE/g extract)**	**TFC (mg QE/g extract)**	**GiA-1 (%w/w)**
Hex	1.33	2.99 ± 0.60	n/d	n/d
DCM	1.10	15.35 ± 0.81	n/d	n/d
EtOAc	0.34	9.10 ± 0.86	2.83 ± 1.48	n/d
MeOH	13.87	27.72 ± 1.10	15.39 ± 2.63	14.95 ± 0.14

n/d = not detected

Table S2. GC/MS phytochemicals profile of the *G. inodorum* extract prepared by hexane.

Compound	RT (min)	Tentative compounds	Chemical formula	CAS no.	MW	Quality (%matching)	Anti-inflammation	Anti-insulin resistance
1	14.329	Dodecanoic acid (Lauric acid)	$C_{12}H_{24}O_2$	000143-07-7	200.18	98	(Yeh et al., 2019), (Roshankhah et al., 2020)	(Tham et al., 2020)
2	17.247	Tetradecanoic acid (myristic acid)	$C_{14}H_{28}O_2$	000544-63-8	228.21	98	(Alonso-Castro et al., 2022)	(Khalil et al., 2021), (Saraswathi et al., 2022)
3	17.68	Neophytadiene	$C_{20}H_{38}$	-	278.3	99	(Arai et al., 2015), (Ratheesh et al., 2022), (Ferdous et al., 2020)	(Fazelipour et al., 2021), (Azemi et al., 2021)
4	17.876	6,10,14-trimethylpentadecan-2-one		000502-69-2	268.28	92	-	-
5	19.339	Palmitic acid	$C_{16}H_{32}O_2$	000057-10-3	256.24	99	induced	induced
6	19.756	9-Octadecenoic acid (oleic acid)	$C_{18}H_{34}O_2$	000112-80-1	282.26	91	(Tian et al., 2017)	(López-Gómez et al., 2020), (Tian et al., 2017)
7	20.148	9,12-Octadecadienoic acid, methyl ester	$C_{19}H_{34}O_2$	000112-63-0	294.26	93	(Zhong et al., 2015), (Ezirim et al., 2019)	(Mahmood et al., 2020), (Nasution et al., 2018)
8	20.205	9,12,15-Octadecatrienoic acid, methyl ester	$C_{19}H_{32}O_2$	007361-80-0	292.24	98	(Amor et al., 2009), (Ujita et al., 2009)	(Li et al., 2012)
9	20.373	Phytol isomer	$C_{20}H_{40}O$		296.31	95	(Islam et al., 2020)	(Elmazar et al., 2013), (Matsuda et al., 2017)
10	20.838	9,12-Octadecadienoic acid (linoleic acid)	$C_{18}H_{32}O_2$	000060-33-3	280.24	99	(Djuricic and Calder, 2021)	(Zaky et al., 2022), (Yoon et al., 2021)
11	21.054	octadecanoic acid (Stearic acid)	$C_{18}H_{36}O_2$	000057-11-4	284.27	99	induced	induced
12	25.871	Squalene	$C_{30}H_{50}$	007683-64-9	410.39	97	(Ibrahim and Naina	(Ganbold et al., 2020)

							Mohamed, 2021)	
13	27.378	β-tocopherol	$C_{28}H_{48}O_2$	000148-03-8	416.37	97	-	-
14	27.466	γ-tocopherol	$C_{28}H_{48}O_2$	007616-22-0	416.37	93	(Reiter et al., 2007)	(Dey et al., 2018), (Zafar et al., 2021)
15	27.659	Octacosane	$C_{28}H_{58}$	000630-02-4	394.45	98	(Okechukwu, 2020)	(Okokon et al., 2022), (Sulaimon et al., 2020)
16	27.955	α-tocopherol (DL)	$C_{29}H_{50}O_2$	010191-41-0	430.38	99	(Reiter et al., 2007)	(Kim et al., 2013), (Pang and Chin, 2019)
17	28.757	Stigmasterol	$C_{29}H_{48}O$	000083-48-7	412.37	94	(Morgan et al., 2021)	(Wang et al., 2017), (Ward et al., 2017)
18	28.877	Octacosane	$C_{28}H_{58}$	000630-02-4	394.45	98	(Okechukwu, 2020)	(Okokon et al., 2022), (Sulaimon et al., 2020)
19	29.094	γ-sitosterol	$C_{29}H_{50}O$	000083-47-6	414.39	98	-	-

Table S3 GC/MS phytochemicals profile of the *G. inodorum* extract prepared by dichloromethane.

**Compound**	**RT (min)**	**Tentative compounds**	**Chemical formula**	**CAS no.**	**MW**	**Quality (%matching)**	**Anti-inflammation**	**Anti-insulin resistance**
1	17.736	Neophytadiene	$C_{20}H_{38}$	-	278.3	99	(Arai et al., 2015), (Ratheesh et al., 2022), (Ferdous et al., 2020)	(Fazelipour et al., 2021), (Azemi et al., 2021)
2	17.832	6,10,14-trimethylpentadecan-2-one	-	000502-69-2	268.28	86	-	-
3	18.176	Phytol	$C_{20}H_{40}O$	-	296.31	89	(Islam et al., 2020)	(Elmazar et al., 2013), (Matsuda et al., 2017)

4	18.633	Palmitic acid, methyl ester	$C_{17}H_{34}O_2$	-	270.26	99	induced	induced
5	19.275	Palmitic acid	$C_{16}H_{32}O_2$	000057-10-3	256.24	99	induced	induced
6	20.276	Linolenic acid, methyl ester	$C_{19}H_{32}O_2$	-	292.24	98	(Pauls et al., 2018), (Kapoor and Huang, 2006)	(Wang et al., 2013), (Gonçalves et al., 2018)
7	20.437	Phytol	$C_{20}H_{40}O$	-	296.31	91	(Islam et al., 2020)	(Elmazar et al., 2013), (Matsuda et al., 2017)
8	20.886	9,12,15-Octadecatrien-1-ol	$C_{18}H_{32}O$	-	264.25	94	(Xia et al., 2018)	(Ali et al., 2020)
9	27.442	β-tocopherol	$C_{28}H_{48}O_2$	000148-03-8	416.37	98	-	-
10	28.027	D, α-tocopherol	$C_{29}H_{50}O_2$	010191-41-0	430.38	97	(Reiter et al., 2007)	(Kim et al., 2013), (Pang and Chin, 2019)
11	28.813	Stigmasterol	$C_{29}H_{48}O$	000083-48-7	412.37	99	(Morgan et al., 2021)	(Wang et al., 2017), (Ward et al., 2017)
12	29.173	γ-sitosterol	$C_{29}H_{50}O$	000083-47-6	414.39	93	-	-

Table S4. GC/MS phytochemicals profile of the *G. inodorum* extract prepared by ethyl acetate.

Compound	RT (min)	Tentative compounds	Chemical formula	CAS no.	MW	Quality (%matching)	Anti-inflammation	Anti-insulin resistance
1	14.329	Dodecanoic acid, methyl ester	$C_{13}H_{26}O_2$	-	214.19	87	(Yeh et al., 2019), (Roshankhah et al., 2020)	(Tham et al., 2020)
2	14.433	Phenol-2,4-bis (1,1-dimethylethyl)	$C_{14}H_{22}O$	-	206.17	91	(George et al., 2018)	(George et al., 2018)
3	16.59	Tetradecanoic acid, methyl ester	$C_{15}H_{30}O_2$	-	242.22	95	(Alonso-Castro et al., 2022)	(Khalil et al., 2021), (Saraswathi et al., 2022)
4	17.736	Neophytadiene	$C_{20}H_{38}$	-	278.3	99	(Arai et al., 2015), (Ratheesh et al., 2022), (Ferdous et al., 2020)	(Fazelipour et al., 2021), (Azemi et al., 2021)
5	17.832	6,10,14-trimethylpentadecan-2-one	-	000502-69-2	268.28	98	-	-
6	17.99	Neophytadiene	$C_{20}H_{38}$	-	278.3	89	(Arai et al., 2015), (Ratheesh et al., 2022), (Ferdous et al., 2020)	(Fazelipour et al., 2021), (Azemi et al., 2021)

7	18.169	Neophytadiene	$C_{20}H_{38}$	-	278.3	89	(Arai et al., 2015), (Ratheesh et al., 2022), (Ferdous et al., 2020)	(Fazelipour et al., 2021), (Azemi et al., 2021)
8	18.633	Pentadecanoic acid, 14-methyl-, methyl ester	$C_{17}H_{34}O_2$	-	270.26	99	-	-
9	19.259	Palmitic acid	$C_{16}H_{32}O_2$	000057-10-3	256.24	99	induced	induced
10	20.421	Phytol	$C_{20}H_{40}O$	-	296.31	87	(Islam et al., 2020)	(Elmazar et al., 2013), (Matsuda et al., 2017)
11	20.509	octadecanoic acid, methyl ester	$C_{19}H_{38}O_2$	-	298.29	95	induced	induced
12	24.717	Eicosane	$C_{20}H_{42}$	-	282.33	94	(Okechukwu, 2020)	-
13	27.442	β-tocopherol	$C_{28}H_{48}O_2$	000148-03-8	416.37	98	-	-
14	28.813	Stigmasterol	$C_{29}H_{48}O$	000083-48-7	412.37	99	(Morgan et al., 2021)	(Wang et al., 2017), (Ward et al., 2017)

Table S5 GC/MS phytochemicals profile of the *G. inodorum* extract prepared by methanol.

Compound	RT (min)	Tentative compounds	Chemical formula	CAS no.	MW	Quality (%matching)	Anti-inflammation	Anti-insulin resistance
1	17.744	Neophytadiene	$C_{20}H_{38}$	-	278.3	97	(Arai et al., 2015), (Ratheesh et al., 2022), (Ferdous et al., 2020)	(Fazelipour et al., 2021), (Azemi et al., 2021)
2	18.642	14-methyl-pentadecanoic acid, methyl ester	$C_{17}H_{34}O_2$	-	270.5	99	-	-
3	20.269	9-octadecanoic acid (E, Z)	$C_{19}H_{36}O_2$	-	296.27	99	-	-

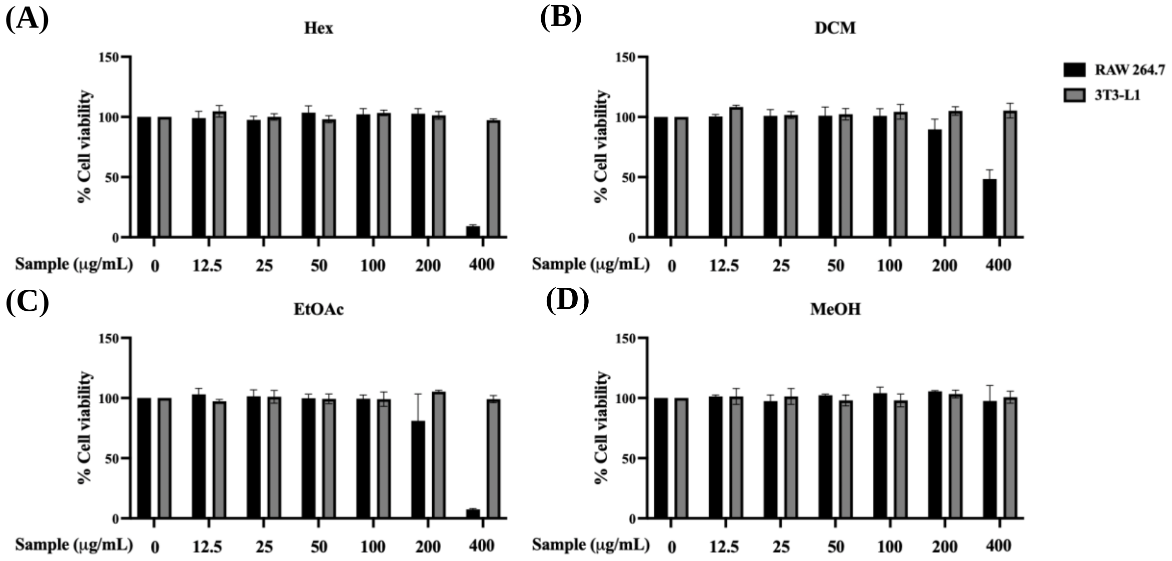

Figure S1 Effects of Hex (A), DCM (B), EtOAc (C) and MeOH (D) on cell viability of RAW 264.7 macrophages and 3T3-L1 mature adipocytes. The cells were treated with various concentrations of samples and each particle (0–800 μg/mL) for 24 hours.

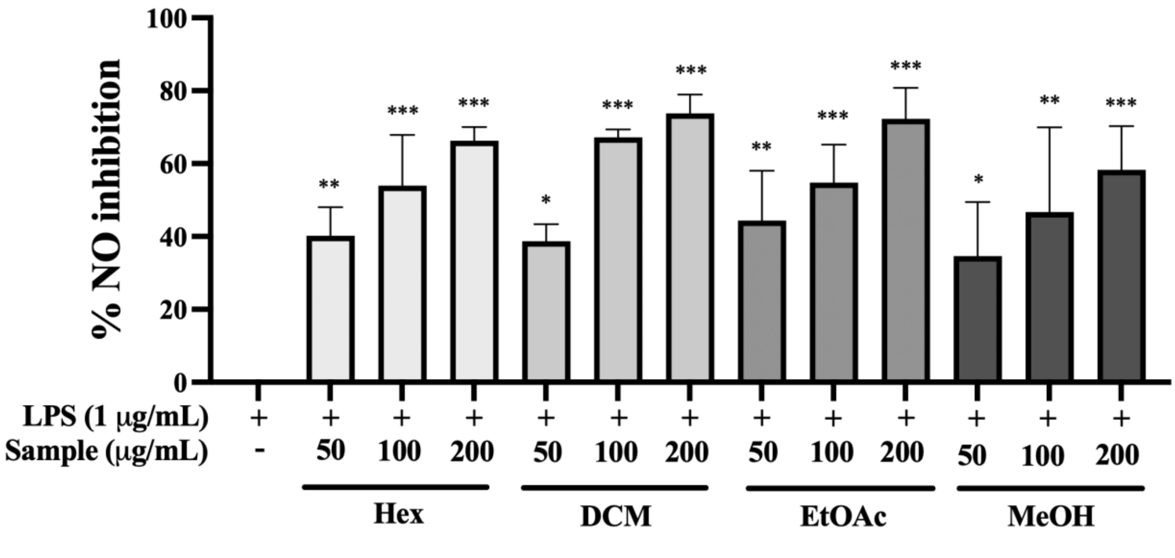

Figure S2 Inhibitory effects of the extracts prepared by hexane (Hex), dichloromethane (DCM), ethyl acetate (EtOAc) and methanol (MeOH) on nitric oxide (NO) production from inflamed RAW 264.7 macrophage cells induced by lipopolysaccharide (LPS). The percentages of NO inhibition are expressed relative to the control with LPS treatment. *, ** and *** represents the levels of *P*-values less than 0.05, 0.01 and 0.001, compared to the LPS-treated control.

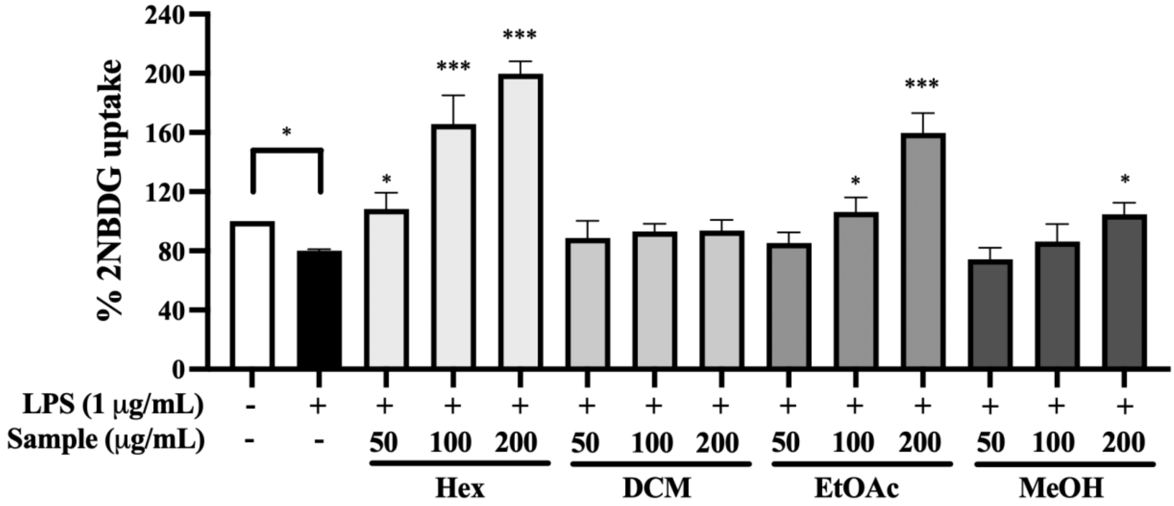

Figure S3 Effect of the extracts prepared by hexane (Hex), dichloromethane (DCM), ethyl acetate (EtOAc) and methanol (MeOH) on LPS- induced insulin resistance on 3T3-L1 adipocytes. The cells were co-treated with 1 μg/mL LPS and the samples at various concentrations for 24 h. The percentages of 2-NBDG uptake are expressed relative to the control without LPS treatment. * and *** represents the levels of *P*-values less than 0.05 and 0.001, compared to the LPS-untreated control.

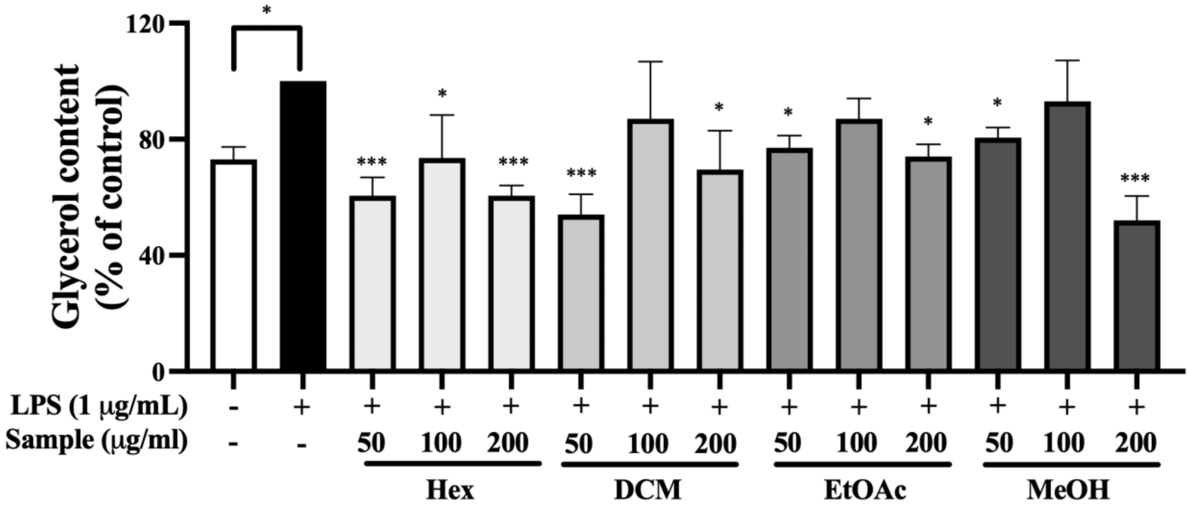

Figure S4 Effect of the extracts prepared by hexane (Hex), dichloromethane (DCM), ethyl acetate (EtOAc) and methanol (MeOH) on glycerol release from LPS- induced insulin resistance on 3T3-L1 adipocytes. The cells were co-treated with 1 μg/mL LPS and the samples at various concentrations for 24 h. The percentages of glycerol release are expressed relative to the control with LPS treatment. * and *** represents the levels of *P*-values less than 0.05 and 0.001, compared to the LPS-untreated control.

References

Ali, A.M.; Gabbar, M.A.; Abdel-Twab, S.M.; Fahmy, E.M.; Ebaid, H.; Alhazza, I.M.; Ahmed, O.M. Antidiabetic Potency, Antioxidant Effects, and Mode of Actions of *Citrus Reticulata* Fruit Peel Hydroethanolic Extract, Hesperidin, And Quercetin in Nicotinamide/Streptozotocin-Induced Wistar Diabetic Rats. *Oxid Med Cell Longev*. **2020**, *2020*, 1730492. https://doi.org/10.1155/2020/1730492

Alonso-Castro, A.J.; Serrano-Vega, R.; Pérez Gutiérrez, S.; Isiordia-Espinoza, M.A.; Solorio-Alvarado, C.R. Myristic Acid Reduces Skin Inflammation and Nociception. *J. Food Biochem.* **2022**, *46*, E14013.

Amor, I.L.-B.; Boubaker, J.; Sgaier, M.B.; Skandrani, I.; Bhouri, W.; Neffati, A.; Kilani, S.; Bouhlel, I.; Ghedira, K.; Chekir-Ghedira, L. Phytochemistry and Biological Activities of Phlomis Species. *J. Ethnopharmacol.* **2009**, *125*, 183–202.

Arai, R.; Nukazawa, K.; Kazama, S.; Takemon, Y. Variation in Benthic Invertebrate Abundance Along Thermal Gradients Within Headwater Streams of a Temperate Basin in Japan. *Hydrobiologia* **2015**, *762*, 55–63.

Azemi, A.K.; Mokhtar, S.S.; Rasool, A.H.G. *Clinacanthus Nutans*: Its Potential Against Diabetic Vascular Diseases. *Braz. J. Pharm. Sci*. **2021**, *56*. https://doi.org/10.1590/S2175-97902020000118838

Dey, P.; Mah, E.; Li, J.; Jalili, T.; Symons, J.D.; Bruno, R.S. Improved Hepatic γ-Tocopherol Status Limits Oxidative and Inflammatory Stress-Mediated Liver Injury in *db/db* Mice with Nonalcoholic Steatohepatitis. *J. Funct. Foods* **2018**, *40*, 670–678.

Djuricic, I.; Calder, P.C. Beneficial Outcomes of Omega-6 and Omega-3 Polyunsaturated Fatty Acids on Human Health: An Update for 2021. *Nutrients* **2021**, *13*, 2421.

Elmazar, M.M.; El-Abhar, H.S.; Schaalan, M.F.; Farag, N.A. Phytol/Phytanic Acid and Insulin Resistance: Potential Role of Phytanic Acid Proven by Docking Simulation and Modulation of Biochemical Alterations. *Plos One* **2013**, *8*, E45638.

Ezirim, C.Y.; Abarikwu, S.O.; Uwakwe, A.A.; Mgbudom-Okah, C.J. Protective Effects of *Anthocleista Djalonensis* A. Chev Root Extracts Against Induced Testicular Inflammation and Impaired Spermatogenesis in Adult Rats. *Mol. Biol. Rep*. **2019**, *46*, 5983–5994. https://doi.org/10.1007/S11033-019-05033-W

Fazelipour, S.; Hadipour Jahromy, M.; Tootian, Z.; Goodarzi, N. Antidiabetic Effects of the Ethanolic Extract of *Allium Saralicum* RM Fritsch on Streptozotocin-Induced Diabetes in A Mice Model. *Food Sci. Nutr.* **2021**, *9*, 4815–4826.

Ferdous, A.; Janta, R.A.; Arpa, R.N.; Afroze, M.; Khan, M.; Moniruzzaman, M. The Leaves of *Bougainvillea Spectabilis* Suppressed Inflammation and Nociception In Vivo Through the Modulation of Glutamatergic, Cgmp, And ATP-Sensitive K+ Channel Pathways. *J. Ethnopharmacol*. **2020**, *261*, 113148.

Ganbold, M.; Ferdousi, F.; Arimura, T.; Tominaga, K.; Isoda, H. New Amphiphilic Squalene Derivative Improves Metabolism of Adipocytes Differentiated from Diabetic Adipose-Derived Stem Cells and Prevents Excessive Lipogenesis. Front. *Cell Dev. Biol*. **2020**, *8*, 577259.

George, L.O.; Radha, H.R.; Somasekariah, B.V. In Vitro Anti-Diabetic Activity and GC-MS Analysis of Bioactive Compounds Present in the Methanol Extract of *Kalanchoe Pinnata*. *NISCAIR-CSIR* **2018**, 1213-1221.

Gonçalves, N.B.; Bannitz, R.F.; Silva, B.R.; Becari, D.D.; Poloni, C.; Gomes, P.M.; Foss, M.C.; Foss-Freitas, M.C. A-Linolenic Acid Prevents Hepatic Steatosis and Improves Glucose Tolerance in Mice Fed a High-Fat Diet. *Clinics* **2018**, *73*.

Ibrahim, N.; Naina Mohamed, I. Interdependence of Anti-Inflammatory and Antioxidant Properties of Squalene–Implication for Cardiovascular Health. *Life* **2021**, *11*, 103. Https://Doi.Org/10.3390/Life11020103

Islam, M.T.; Ayatollahi, S.A.; Zihad, S.N.K.; Sifat, N.; Khan, M.R.; Paul, A.; Salehi, B.; Islam, T.; Mubarak, M.S.; Martins, N. Phytol Anti-Inflammatory Activity: Pre-Clinical Assessment and Possible Mechanism of Action Elucidation. *Cell. Mol. Biol*. ***2020***, *66*, 264–269.

Khalil, A.S.M.; Giribabu, N.; Yelumalai, S.; Shahzad, H.; Kilari, E.K.; Salleh, N. Myristic Acid Defends Against Testicular Oxidative Stress, Inflammation, Apoptosis: Restoration of Spermatogenesis, Steroidogenesis in Diabetic Rats. *Life Sci*. **2021**, *278*, 119605.

Kim, D.Y.; Kim, J.; Ham, H.J.; Choue, R. Effects Of D-A-Tocopherol Supplements on Lipid Metabolism in A High-Fat Diet-Fed Animal Model. *Nutr. Res. Pract*. **2013**, *7*, 481–487.

Li, L.; Wang, Q.; Yang, Y.; Wu, G.; Xue-Lei, X.; Aisa, H. Chemical Components and Antidiabetic Activity of Essential Oils Obtained by Hydrodistillation and Three Solvent Extraction Methods from *Carthamus Tinctorius* L. *Acta Chromatogr*. **2012**, *24*, 653. Https://Doi.Org/10.1556/Achrom.24.2012.4.11

López-Gómez, C.; Santiago-Fernández, C.; García-Serrano, S.; García-Escobar, E.; Gutiérrez-Repiso, C.; Rodríguez-Díaz, C.; Ho-Plágaro, A.; Martín-Reyes, F.; Garrido-Sánchez, L.; Valdés, S. Oleic Acid Protects Against

Insulin Resistance by Regulating the Genes Related to the PI3K Signaling Pathway. *J. Clin. Med*. **2020**, *9*, 2615.

Mahmood, R.; Kayani, W.K.; Ahmed, T.; Malik, F.; Hussain, S.; Ashfaq, M.; Ali, H.; Rubnawaz, S.; Green, B.D.; Calderwood, D. Assessment of Antidiabetic Potential and Phytochemical Profiling of Rhazya Stricta Root Extracts. BMC Complement. *Med. Ther.* **2020**, *20*, 1–17.

Matsuda, H.; Suzuki, D.; Asakura, M.; Ooi, S.; Saitoh, R.; Otokozawa, R., Shirai, T. Effects of Dietary Phytol on Glucose Uptake and Insulin Secretion In Vitro and In Vivo. *Food Nutr. Current Res*. **2018**, *1*, 29-37.

Morgan, L.V.; Petry, F.; Scatolin, M.; De Oliveira, P.V.; Alves, B.O.; Zilli, G.A.L.; Volfe, C.R.B.; Oltramari, A.R.; De Oliveira, D.; Scapinello, J. Investigation of the Anti-Inflammatory Effects of Stigmasterol in Mice: Insight Into Its Mechanism of Action. *Behav. Pharmacol*. **2021**, *32*, 640–651.

Nasution, R.; Fitrah, C.N.; Helwati, H.; Murniana, A.B.; Cutchamzurni, C. Antidiabetes Activities Extract Hexane from the Peels of *Artocarpus Camansi* Blanco Fruit. *Asian J. Pharm. Clin. Res*. **2018**, *11*, 12–7.

Okechukwu, P.N. Evaluation of Anti-Inflammatory, Analgesic, Antipyretic Effect of Eicosane, Pentadecane, Octacosane, and Heneicosane. *Asian J. Pharm. Clin. Res.* **2020**, 29–35. Https://Doi.Org/10.22159/Ajpcr.2020.V13i4.36196

Okokon, J.E.; Etuk, I.C.; Thomas, P.S.; Drijfhout, F.P.; Claridge, T.D.W.; Li, W.-W. In Vivo Antihyperglycaemic and Antihyperlipidemic Activities and Chemical Constituents of S*olanum Anomalum*. *Biomed. Pharmacother. Biomedecine Pharmacother.* **2022**, *151*, 113153. https://doi.org/10.1016/J.Biopha.2022.113153

Pang, K.-L.; Chin, K.-Y. The Role of Tocotrienol in Protecting Against Metabolic Diseases. *Molecules* **2019**, *24*, 923.

Pauls, S.D.; Rodway, L.A.; Winter, T.; Taylor, C.G.; Zahradka, P.; Aukema, H.M. Anti-Inflammatory Effects of α-Linolenic Acid in M1-Like Macrophages are Associated with Enhanced Production of Oxylipins From α-Linolenic and Linoleic Acid. *J. Nutr. Biochem*. **2018**, *57*, 121–129.

Ratheesh, M.; Sunil, S.; Sheethal, S.; Jose, S.P.; Sandya, S.; Ghosh, O.S.N.; Rajan, S.; Jagmag, T.; Tilwani, J. Anti-Inflammatory and Anti-COVID-19 Effect of a Novel Polyherbal Formulation (Imusil) Via Modulating Oxidative Stress, Inflammatory Mediators and Cytokine Storm. *Inflammopharmacology* **2022**, *30*, 173–184.

Reiter, E.; Jiang, Q.; Christen, S. Anti-Inflammatory Properties of α-And γ-Tocopherol. *Mol. Aspects Med*. **2007**, *28*, 668–691.

Roshankhah, S.; Abdolmaleki, A.; Salahshoor, M.R. Anti-Inflammatory, Anti-Apoptotic, and Antioxidant Actions of Middle Eastern Phoenix Dactylifera Extract on Mercury-Induced Hepatotoxicity In Vivo. *Mol. Biol. Rep.* **2020**, *47*, 6053–6065.

Saraswathi, V.; Kumar, N.; Ai, W.; Gopal, T.; Bhatt, S.; Harris, E.N.; Talmon, G.A.; Desouza, C.V. Myristic Acid Supplementation Aggravates High Fat Diet-Induced Adipose Inflammation and Systemic Insulin Resistance in Mice. *Biomolecules* **2022**, *12*, 739.

Sulaimon, L.A.; Anise, E.O.; Obuotor, E.M.; Samuel, T.A.; Moshood, A.I.; Olajide, M.; Fatoke, T. In Vitro Antidiabetic Potentials, Antioxidant Activities and Phytochemical Profile of African Black Pepper (*Piper Guineense*). *Clin. Phytoscience* **2020**, *6*, 1–13.

Tham, Y.Y.; Choo, Q.C.; Muhammad, T.S.T.; Chew, C.H. Lauric Acid Alleviates Insulin Resistance by Improving Mitochondrial Biogenesis in THP-1 Macrophages. *Mol. Biol. Rep.* **2020**, *47*, 9595–9607.

Tian, X.; Seluanov, A.; Gorbunova, V. Molecular Mechanisms Determining Lifespan in Short-And Long-Lived Species. *Trends Endocrinol. Metab*. **2017**, *28*, 722–734.

Ujita, M.; Nagayama, H.; Kanie, S.; Koike, S.; Ikeyama, Y.; Ozaki, T.; Okumura, H. Carbohydrate Binding Specificity of Recombinant Human Macrophage B-Glucan Receptor Dectin-1. *Biosci. Biotechnol. Biochem*. **2009**, *73*, 237–240.

Wang, D.Q.; Liu, X.L.; Rong, Q.F.; Han, L.; Zhao, N.Q. Alpha-Linolenic Acid Improves Insulin Sensitivity in Obese Patients. *Zhonghua Yi Xue Za Zhi* **2013**, *93*, 132–134.

Wang, J.; Huang, M.; Yang, J.; Ma, X.; Zheng, S.; Deng, S.; Huang, Y.; Yang, X.; Zhao, P. Anti-Diabetic Activity of Stigmasterol from Soybean Oil by Targeting Tte GLUT4 Glucose Transporter. *Food Nutr. Res*. **2017**, *61*, 1364117.

Ward, M.G.; Li, G.; Barbosa-Lorenzi, V.C.; Hao, M. Stigmasterol Prevents Glucolipotoxicity Induced Defects in Glucose-Stimulated Insulin Secretion. *Sci. Rep*. **2017**, *7*, 1–13.

Xia, M.; Liu, L.; Qiu, R.; Li, M.; Huang, W.; Ren, G.; Zhang, J. Anti-Inflammatory and Anxiolytic Activities of *Euphorbia Hirta* Extract in Neonatal Asthmatic Rats. *AMB Express* **2018**, *8*, 1–11.

Yeh, C.-F.; Chuang, T.-Y.; Hung, Y.-W.; Lan, M.-Y.; Tsai, C.-H.; Huang, H.-X.; Lin, Y.-Y. Soluble Epoxide Hydrolase Inhibition Enhances Anti-Inflammatory and Antioxidative Processes, Modulates Microglia Polarization, and Promotes Recovery After Ischemic Stroke. *Neuropsychiatr. Dis. Treat* **2019**, *15*, 2927.

Yoon, S.-Y.; Ahn, D.; Hwang, J.Y.; Kang, M.J.; Chung, S.J. Linoleic Acid Exerts Antidiabetic Effects by Inhibiting Protein Tyrosine Phosphatases Associated with Insulin Resistance. *J. Funct. Foods* **2021**, *83*, 104532.

Zafar, H.; Mirza, I.A.; Hussain, W.; Fayyaz, M. Comparative Efficacy of Tocotrienol and Tocopherol for Their Anti Diabetic Effects. *Biomed. J. Sci. Tech. Res.* **2021**, *38*, 30835–30840.

Zaky, A.S.; Kandeil, M.; Abdel-Gabbar, M.; Fahmy, E.M.; Almehmadi, M.M.; Ali, T.M.; Ahmed, O.M. The Antidiabetic Effects and Modes of Action of The *Balanites Aegyptiaca* Fruit and Seed Aqueous Extracts in NA/STZ-Induced Diabetic Rats. *Pharmaceutics* **2022**, *14*, 263.

Zhong, R.-F.; Xu, G.-B.; Wang, Z.; Wang, A.-M.; Guan, H.-Y.; Li, J.; He, X.; Liu, J.-H.; Zhou, M.; Li, Y.-J. Identification of Anti-Inflammatory Constituents from Kalimeris Indica with UHPLC-ESI-Q-TOF-MS/MS and GC–MS. *J. Ethnopharmacol*. **2015**, *165*, 39–45.
